# No added cost: Emotion recognition in co‐occurring ADHD and ASD

**DOI:** 10.1111/bjc.70056

**Published:** 2026-04-12

**Authors:** Irene Sophia Plank, Kaltrina Krasniqi, Alexandra Pior, Julia Nowak, Christine M. Falter‐Wagner

**Affiliations:** ^1^ Department of Psychiatry and Psychotherapy, LMU University Hospital LMU Munich Munich Germany; ^2^ Department of Sociology, Faculty of Behavioral and Social Sciences University of Groningen Groningen Netherlands; ^3^ Department of Psychology, Faculty of Psychology and Neuroscience Maastricht University Maastricht Netherlands

**Keywords:** affect recognition, ASD, attention‐deficit/hyperactivity disorder, autism, emotion recognition, facial emotion recognition, neurodevelopmental disorders

## Abstract

**Objectives:**

Facial emotion recognition (FER) is fundamental for social interaction, yet this ability is often impaired in attention‐deficit/hyperactivity disorder (ADHD) or autism spectrum disorder (ASD). While research has documented these difficulties, two key questions remain: Are these deficits specific to recognising emotions or do they reflect broader challenges in face processing? Does the increasingly documented co‐occurrence of ADHD and ASD exacerbate these difficulties?

**Methods:**

We compared emotion discrimination thresholds (EDT) in *N* = 92 participants aged 17–45 years (mean = 28.5, SD = 7.2), including 22 autistic‐only, 24 ADHD‐only, 22 co‐occurring ADHD+ASD and 24 typically‐developing individuals. We measured EDT with faces gradually changing from a neutral to an emotional expression (angry, fearful, happy or sad) and designed a non‐emotional control task. Using eye tracking, we ruled out potential differences in visual attention to facial features between groups.

**Results:**

We showed a specific disadvantage in emotion in the clinical groups, with increased EDT in all three clinical groups, with effect sizes ranging from .43 for co‐occurring ADHD+ASD to .77 for autistic‐only. Crucially, emotion recognition deficits were not amplified by the co‐occurrence of ADHD and ASD, when considering performance in the control task.

**Conclusions:**

Our study possibly suggests equal mechanisms underlying reduced FER in ASD and ADHD. This mechanism should be identified to develop targeted interventions addressing social cognitive challenges in ASD and ADHD, improving social integration and well‐being. Clinicians should recognise that FER difficulties are shared across both conditions, since a primary association with ASD may lead to unwarranted assumptions of ASD co‐occurrence in ADHD.

**Practitioner points**

- Deficits in social cognition, including facial emotion recognition, are a symptom of ASD that persists in adults.
- While not a symptom of ADHD, adults with ADHD nonetheless show a similar deficit for recognising emotions in facial expressions.
- This shared deficit across neurodevelopmental disorders could suggest equal mechanisms.
- Thus, including interventions to support patients in their emotion recognition abilities is as important for adults with ADHD as it is for adults with ASD.

## INTRODUCTION

Despite differences in the nosology, autism and attention deficit/hyperactivity disorder (ADHD) show some overlapping genetic vulnerability and behavioural phenomenology in selected areas (Antshel & Russo, 2019). In ICD‐11, autism is diagnosed on the basis of difficulties in social interaction and communication as well as repetitive and restricted behaviours, while ADHD is specified by inattention and/or hyperactivity and impulsivity (World Health Organization, 2019). The diagnostic criteria in the DSM‐V‐TR are similar to those in ICD‐11: for ASD, they are persistent deficits in social communication and interaction across contexts and restricted, repetitive patterns of behaviour, interests or activity; for ADHD, the criteria in DSM‐V‐TR are inattention and/or hyperactivity and impulsivity (American Psychiatric Association, 2022). There is a high rate of co‐occurrence between these conditions, with about 28% of autistic adults also being diagnosed with ADHD (Lai et al., 2019).

One area of commonalities in the presentation of ADHD and autism is difficulties recognising emotions associated with facial expression, with emotion recognition difficulties reported in reviews and meta‐analyses of children, adolescents and adults with ADHD (Arango‐Tobón et al., 2023; Bora & Pantelis, 2016; Borhani & Nejati, 2018; Shepard et al., 2025) or ASD (Harms et al., 2010; Leung et al., 2022; Lozier et al., 2014; Masoomi et al., 2025; Uljarevic & Hamilton, 2013; Yeung, 2022). In addition to assessing facial emotion recognition (FER) in autism, the meta‐analysis by Yeung (2022) also looked at effects comparing FER in autistic individuals and individuals with other clinical conditions, including ADHD, finding significantly lower FER abilities in ASD than in other conditions. Similarly, Bora and Pantelis (2016) also found more pronounced social cognitive deficits in ASD compared with ADHD. However, in both meta‐analyses, almost all studies included had investigated children or adolescents as participants. A recent systematic review examining studies on children and adolescents with ASD and ADHD found impairments in emotion recognition in both conditions, with some studies indicating a more pronounced deficit in ASD and co‐occurrence possibly exacerbating challenges with emotion recognition (Lievore et al., 2025). The review also reports differences in the development of emotion recognition difficulties. While children with ASD show emotion recognition difficulties early in life, children and adolescents with ADHD seem to develop deficits as they age. Despite the early onset, a meta‐analysis found that FER deficits increase with age in ASD (Lozier et al., 2014), while one review suggests that FER in ADHD might improve with age (Olaya‐Galindo et al., 2023).

Research comparing FER in adults with autism and ADHD is scarce. Gonzalez‐Gadea and colleagues (Gonzalez‐Gadea et al., 2013) used the reading the mind in the eyes test (Baron‐Cohen et al., 1997) and found no differences between adults with ASD and adults with ADHD; however, they also found no differences between the clinical groups and typically‐developing adults. In another study, adult participants were asked to judge whether consecutively presented facial expressions portrayed a specific target emotion (Helfer et al., 2021). Autistic adults and adults with ADHD had increased reaction times compared to typically‐developing adults without any psychiatric diagnoses, with no significant difference between participants with autism or ADHD (Helfer et al., 2021). However, all three groups showed comparable error rates in this task, possibly suggesting a difference in speed‐accuracy trade‐off or important confounding factors like slower motor responses.

The speed‐accuracy trade‐off occurs whenever tasks collect both response times and accuracy scores, leading to longer response times increasing the probability of correct responses (Vandierendonck, 2017). People with neurodevelopmental disorders may employ different strategies to manage this speed‐accuracy trade‐off (Helfer et al., 2021). To control for such differences, previous studies have attempted to determine the discrimination threshold of participants. Participant‐specific emotion discrimination thresholds (EDT) capture the amount of information that a participant needs to correctly recognise the emotion associated with facial expressions. EDTs can be assessed using videos created by morphed images, with studies showing the potential for using such a morphing task to measure EDT in people with neurodevelopmental disorders to mitigate different strategies in the speed‐accuracy trade‐off (Jusyte et al., 2017; Schönenberg et al., 2019).

An important possible confound of previous results regarding FER is that individuals with ADHD or autism exhibit increased reaction times on a variety of tasks (Morrison et al., 2018; Pievsky & McGrath, 2018). This increase in reaction time aggravates the interpretation of existing research showing differences in reaction times on emotion recognition tasks since these differences could be traced back to motor or sensory processing unrelated to emotion recognition itself. For instance, Helfer et al. (2021) found the same pattern of increased response times but similar error rates in adults with autism or ADHD compared to typically‐developing adults with no psychiatric diagnoses not only for the previously mentioned emotion recognition task but also for a similar gender recognition task, where participants indicated the gender of consecutively presented faces. Thus, in the current study, we introduce a paradigm that complements an emotional face‐morphing task measuring EDT with a control task where a human face with an emotionally neutral expression morphs into the face of another animal species; thus, measuring species discrimination threshold (SDT). In contrast to existing studies investigating differences in FER without a control task leaving it unclear whether differences are due to general processing differences or specific to emotion recognition, the design of the current study will allow us to distinguish general differences between the groups from differences in recognising emotions based on facial expressions (Plank et al., 2022).

Moreover, the current study includes eye‐tracking in addition to behavioural performance indices for reasons explicated in the following. Faces are visually rich and highly salient stimuli that give us an important first impression of a person and their affective state. They contain several landmarks that can capture our attention, for example, the eyes and the mouth. The function of recognising emotions from faces may be influenced by which landmark captures one's attention. For instance, smiles are associated with raised cheeks and lips, while anger is associated with lowered brows (Hamm et al., 2011). Eye movements are an important source of information for attention (Vehlen et al., 2021) and can be used to assess focus on different facial landmarks (Kirchner et al., 2011). Some research has suggested that differences in face processing in autistic individuals may stem from differences in attention to faces and other social stimuli (Cuve et al., 2018; Reisinger et al., 2020). Specifically for fixations during FER, studies have found fewer fixations in general (Reisinger et al., 2020), less fixation time spent on the eye region (Bours et al., 2018; Hernandez et al., 2009) or no differences in relative fixation time (Kirchner et al., 2011) between autistic and non‐autistic participants. Additionally, autistic adults may produce fewer saccades between facial landmarks compared to non‐autistic adults when processing faces (Yi et al., 2014). There is less research on individuals with ADHD, but the existing work suggests less fixation time spent on the eye region during FER (Airdrie et al., 2018; Bozkurt et al., 2024). If people with neurodevelopmental disorders focus on different facial landmarks compared to typically‐developing participants, this could offer mechanistic explanations for differences in EDT. Therefore, it is important to assess gaze patterns during FER to investigate attentional differences between typically‐developing and neurodiverse groups.

In this preregistered study, we used two morphing tasks to assess how much information adults with ADHD, autistic adults and typically‐developing adults without any psychiatric diagnoses need to correctly recognise emotions and species, respectively. We used eye tracking to investigate differences in overt attention as measured by gaze fixations to facial landmarks and saccades between them. We hypothesise that typically‐developing adults have a lower EDT, compared to both adults with ADHD and autistic adults, and explore differences in EDT between adults with ASD and adults with ADHD. We expect that these differences in EDT persist when taking SDT measured by the control task into account. This pattern would indicate that typically‐developing adults need less information to correctly recognise associated emotions in facial expressions rather than show increased processing speed or differences in confidence levels. Furthermore, we hypothesise that fixation durations to areas of interest in the faces, specifically the eyes, forehead, mouth and nose, differ between the four groups (Airdrie et al., 2018; Bours et al., 2018; Bozkurt et al., 2024; Hernandez et al., 2009). Last, we hypothesise that typically‐developing adults produce more saccades between areas of interest than autistic adults (Yi et al., 2014). To capture possible differences in salient features, we not only explore differences in fixations to the eyes (Bours et al., 2018; Hernandez et al., 2009) but also compare where groups fixated first, arguably the feature capturing their immediate attention, and where they fixated right before they made their decision which possibly indicates the feature driving this decision (first and last fixations). Last, we include an exploratory group of adults with co‐occurring ADHD and autism to derive mechanistic hypotheses for targeted future research.

## MATERIALS AND METHODS

The here‐presented tasks are part of a larger research project approved by the Ethics committee of the Medical Faculty, LMU Munich (reference number: 22‐0561), and were carried out in accordance with the Declaration of Helsinki (World Medical Association, 2024). The study was preregistered on OSF: https://doi.org/10.17605/OSF.IO/BM7TE.

### Participants

The here‐presented study was part of a larger research project for which we planned to analyse the data of at least *N* = 66 participants, each *n* = 22 with ASD, ADHD and without any psychiatric diagnoses (comparison group, COMP). The sample size was determined based on a power analysis computed with PANGEA v0.2 (Westfall, 2016) with two population‐level predictors (emotion and group) and two group‐level effects (participants nested in group and trials nested in emotion). We chose the sample size to reach a power of 80% for a medium effect (*d* = .45) between two of the groups.

All participants were between 16 and 45 years of age with no neurological diagnoses, their German skills were sufficient to understand the instructions, they had an IQ of at least 70 and either unimpaired or corrected‐to‐normal vision. All participants and any legal guardians provided written informed consent. COMP participants were not currently taking any psychotropic medication. We confirmed that autistic participants in the ASD group had no ADHD diagnosis and vice versa based on existing diagnostic certificates but did not perform a diagnostic evaluation ourselves. Furthermore, we included an exploratory sample of adults with ADHD and ASD (ADHD+ASD group). Some participants performed another task in a magnetic resonance imaging (MRI) scanner and had to fulfil MRI safety criteria. We excluded participants with acute suicidality as assessed by the Beck Depression Inventory (BDI, Hautzinger et al., 1994) or who reported self‐ or other‐harming behaviour as part of our risk management control and due to the negative valence of many of our stimuli. We asked a subsample of 45 autistic adults about their language preferences to align our reporting with those preferences (Bottema‐Beutel et al., 2021; Buijsman et al., 2022; Bury et al., 2022). Since options were similarly popular (person‐first: 37.78%, identity‐first: 40.00%, no preference: 22.22%), we use both throughout the manuscript.

In total, we recruited 108 participants from 2023 to 2025 of which nine were to pilot the tasks, two were excluded due to acute suicidality and three due to a change in diagnostic status (two datasets excluded due to participant report, one due to clinician reassessment). Specifically, all data was collected from February 2023 to January 2025. After additionally excluding two autistic participants with more than ⅓ of the trials answered inaccurately, we analysed 92 participants (22 ADHD+ASD, 24 ADHD, 22 ASD, 24 COMP) for the behavioural analysis of this paper. For the analysis of the eye‐tracking data, additional quality checks as described below led to a slightly smaller sample than preregistered (18 ASD, 16 ADHD, 22 ADHD+ASD, 21 COMP). Groups did not differ in age or IQ estimate according to Bayesian ANOVAs and were comparable in gender distribution according to a Bayesian contingency table (see Table 1). Participants received monetary compensation or study credit points. Furthermore, some of our participants received compensation for travel expenses. We took self‐reported gender and age into consideration during recruitment to ensure that there are no differences between the groups.

**TABLE 1:** Socio‐demographic and questionnaire values, showing means and standard deviations as well as the range in parentheses.

Measurement	ADHD+ASD	ADHD	COMP	ASD	Log(BF)
Age	30.68 ± 8.11 (17 to 44)	26.92 ± 7.19 (17 to 43)	27.42 ± 5.65 (21 to 42)	29.14 ± 7.57 (20 to 45)	−1.449
ASRS‐v1.1	46.64 ± 9.48 (32 to 67)	43.38 ± 12.22 (16 to 62)	25.04 ± 8.24 (13 to 42)	33.36 ± 8.32 (14 to 52)	19.702*
Education	4.00 ± 1.35 (1 to 5)	3.48 ± 1.12 (1 to 5)	4.33 ± .82 (2 to 5)	3.68 ± 1.09 (2 to 5)	.811
Gender	3—12—7	2—9—13	0—11—13	0—11—11	−3.980
IQ estimate	113.23 ± 11.39 (91 to 133)	108.00 ± 11.66 (84 to 130)	109.90 ± 9.41 (92 to 131)	114.25 ± 15.04 (78 to 144)	−1.374
RADS‐R	145.86 ± 34.05 (85 to 201)	93.75 ± 41.37 (18 to 148)	44.58 ± 35.02 (9 to 142)	147.59 ± 38.70 (55 to 203)	28.832*
TAS‐20	56.45 ± 10.55 (37 to 72)	50.29 ± 12.08 (25 to 74)	39.08 ± 9.46 (26 to 63)	62.59 ± 6.56 (48 to 80)	19.137*

*Note*: For gender, we report counts in the following order: Diverse/agender/non‐binary—female—male. Education was operationalised as the highest level of education, ranging from 1 = “no educational or vocational training” to 5 = “University degree.” Asterisk indicates at least moderate evidence in favour of a group difference, corresponding to a log‐transformed Bayes Factor larger than 3.

Abbreviations: ASRS, Adult ADHD Self‐Report Scale; RADS‐R, Ritvo Autism Diagnostic Scale—Revised; TAS, Toronto Alexithymia Scale.

### Experimental procedure

Participants performed four behavioural tasks, and some performed an additional MRI task. We counterbalanced the order of the behavioural tasks with the FER and the Facial Species Recognition (FSR) task always performed consecutively. We report the results of the other tasks elsewhere (Plank, Nowak, et al., 2025; Plank, Pior, et al., 2025; Plank, Yurova, Pior, Nowak, Bierlich, et al., 2025; Plank, Yurova, Pior, Nowak, Shi, et al., 2025). We used a LiveTrack Lightning eye tracker (Cambridge RS, sampling rate 500 Hz) with a headrest during the behavioural tasks, which were performed in a testing chamber with consistent lightning conditions on a Lenovo Legion Pro 5 laptop (resolution: 2560 × 1600 pixel, size: 344 × 215 mm, resolution: 60 Hz), placed 57 cm in front of the participants. Preceding each task, we calibrated the eye tracker individually to the participant's position. We only included data of participants with at least one eye with an accuracy below .5° in the analysis of the eye‐tracking data.

We collected socio‐demographic data, including highest level of education ranging from 1 = “no educational or vocational training” to 5 = “University degree”, and self‐reported assessments of depressive symptoms (BDI, Hautzinger et al., 1994) as well as performed intelligence assessments (MWT‐B, Lehrl, 1999; CFT 20‐R, Weiß, 2006) as part of the evaluation of the inclusion criteria directly after obtaining informed, written consent. Between the tasks, participants filled out the Adult ADHD Self‐Report Scale (ASRS‐v1.1, Kessler et al., 2005) and the Ritvo Autism Diagnostic Scale—Revised (RADS‐R, Rausch et al., 2024), while they filled out the Toronto Alexithymia Scale (TAS‐20, Popp et al., 2008) at the very end of all tasks. The TAS‐20 was collected to explore associations between EDT and alexithymia, since some studies suggest FER deficits in ASD may be caused by co‐occurring alexithymia (Bird & Cook, 2013; Cook et al., 2013; but see also Kirsch et al., 2025). Data collection took between two and four hours, depending on whether participants also performed the task in the MRI scanner.

### Stimulus material

We created a picture series showing a gradual and continuous change in faces to assess discrimination thresholds for emotions and species. For the FSR task, we used already existing stimuli (Plank et al., 2022), but for the FER task, we created new stimuli. We selected 21 female presenting and 22 male presenting identities from the Karolinska Directed Emotional Faces (KDEF) database, allowing us to cut out the face without removing relevant information for the emotional expression (Lundqvist et al., 1998). For each identity, we processed images with an emotionally neutral, angry, fearful, happy and sad facial expression photographed frontally in GIMP *2.10.32* (The GIMP Development Team, 2022) by cutting out the face, grey scaling it and placing it on a black background. Then, the emotionally neutral faces were morphed with the same face showing an emotion with Winmorph© *3.01*, creating 300 pictures showing the gradual and continuous change from the start to the end image (see Figure 1). We evaluated all newly created morphed stimuli that passed an initial visual inspection using an online survey generated and hosted on SoSci (Leiner, 2019). The resulting stimuli had an average naturalness rating of 70.50, and naturalness ratings did not differ between emotions, gender of the stimulus or their interaction (see S1). Please consult the Supplementary Materials S1 for a list of all KDEF IDs used. We created dynamic areas of interest (AOI, see Figure 1) capturing the eye, forehead, mouth and nose region of the presented faces in the FER task based on landmarks extracted with OpenFace (Baltrusaitis et al., 2018).

**FIGURE 1:**
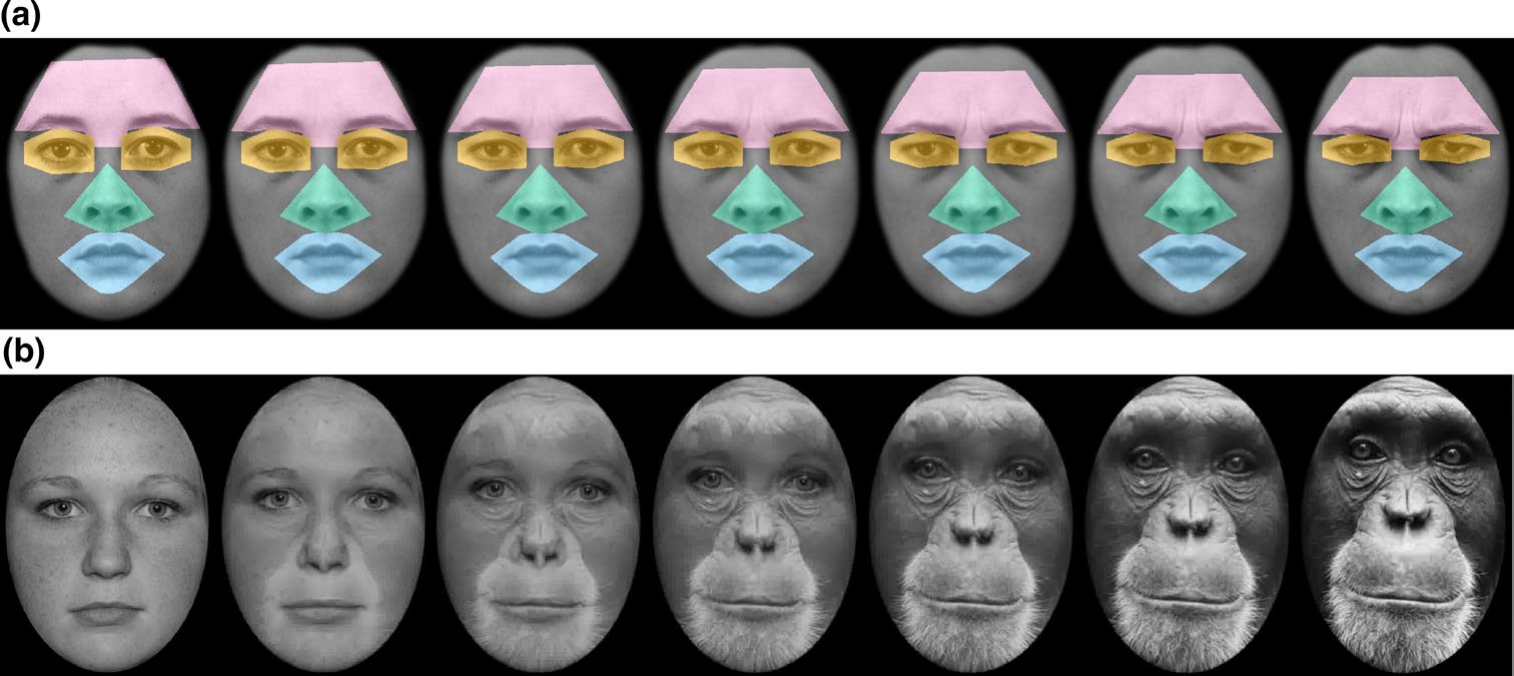
Example still images of both tasks. Subfigure (a) shows still images of the FER task, specifically from a video showing KDEF identity AF14 where the emotionally neutral expression gradually and continually changes into an angry facial expression with overlays showing the dynamic areas of interest (AOIs). Subfigure (b) shows still images from a video where the human face with an emotionally neutral expression gradually and continually changes into an ape.

### Experimental tasks

Both tasks were programmed in Psychtoolbox *3.0.18* (Brainard, 1997; Kleiner et al., 2007) such that participants watched 5‐s‐long videos showing faces slowly and gradually changing. Faces were presented with an approximate height of 1000 pixels and a width of 800 pixels, corresponding to visual angles of 13.45° and 10.41°, respectively. We asked participants to stop the videos as soon as they had recognised either the emotion in the FER task or the species in the FSR task into which the face changed. Participants did not see the final frame after stopping the video but immediately had to choose the emotion or species from four alternatives. They had eight seconds to decide and confirm their choice by key press. The emotions included in the FER task were anger, fear, happiness and sadness (each 16 trials), and the species included in the FSR task were apes, cats, dogs and lions (each four trials). Trial order was pseudo‐randomised so that participants never encountered the same emotion or species more than three times in a row. The FER task took no longer than 14 min, while the FSR task took 4 min at most.

### Preprocessing

We computed the discrimination threshold for each trial by dividing the number of frames before the participants stopped the video by the total number of frames. We only included discrimination thresholds of correctly answered trials in the main analysis. We classified gaze patterns into blinks, saccades and fixations based on the algorithms by Nyström and Holmqvist (2010), using an implementation provided by Niehorster et al. (2015) with the default settings and merging glissades with the saccades. If possible, we averaged the signal of the left and the right eye to increase precision (Cui & Hondzinski, 2006; Hooge et al., 2019). We excluded data of participants who blinked on more than 1/3 of the samples, data of all participants who were excluded from the behavioural analysis as well as data of one participant with ADHD because they had fewer than 64 relevant fixations and saccades, as well as first and last AOIs for fewer than two‐thirds of the trials. This results in the analysis of eye tracking data of 78 participants.

### Analyses

We constructed Bayesian linear (mixed) models with sum‐coded categorical predictors using the brms package (Bürkner, 2017). Group‐level effects were chosen based on Barr (2013) and van Doorn et al. (2023). We followed the guidelines for a Bayesian workflow provided by Schad et al. (2021). For some models, posterior fit was suboptimal and improved by aggregating scores (see Supplementary Materials).

We used two approaches to draw inferences based on our models. For specific hypotheses, we assessed the distribution of the estimated parameters of interest of one model with brms::hypothesis() and an alpha of 5%. For some effects, we additionally performed equivalence tests based on the region of practical equivalence (ROPE) as proposed by Kruschke (2018) allowing us to assess not only differences but also equivalence between groups. For exploratory models, we additionally performed model comparison between models with varied population‐level but same group‐level effects using Bayes Factors based on marginal likelihood estimates as computed by the bridgesampling package (Gronau et al., 2020). To measure the strength of evidence for one model relative to another, we used the log‐transformed Bayes Factor (BF, Lakens et al., 2020) and interpreted it according to Jeffrey's scheme as implemented in the effectsize package (Ben‐Shachar et al., 2020), resulting in a threshold of log(3) for a moderate effect. The stability of our model comparison across priors was confirmed by sensitivity analyses with widened and narrowed standard deviations of our chosen priors (see Supplementary Materials).

#### Behavioural analyses

We assessed the hypotheses regarding the aggregated EDT with a Gaussian linear mixed model (LMM) with group, emotion and their interaction on the population‐level and per participant intercepts on the group‐level. To explore whether differences between groups are stable when taking the SDT into account, we modified the model by adding the scaled SDT as a continuous population‐level effect instead of the group‐level intercept. Following reviewer suggestions, we computed an additional model to ensure the stability of our results, which excluded two autistic participants who watched all videos to completion and included age as a covariate. Additionally, we used a Bayesian ANOVA to compare ranked SDT. To explore differences in error rates, we computed an LMM with a Bernoulli distribution and the population‐level effects group, emotion and their interaction. On the group level, we modelled intercepts and slopes for emotions for the participants as well as intercepts and slopes for the group for the videos. Standardised effect sizes were computed for credible effects by dividing the effect by the sum of all variances (Hedges, 2007). Furthermore, we used Bayesian correlations to explore the relationship between alexithymia as measured by the TAS‐20 (Popp et al., 2008) with EDT within groups.

#### Eye tracking analyses

We analysed dwell times with an LMM including groups, emotions and AOIs with a hurdle lognormal distribution, which is similar to a lognormal distribution but allows for zeros in case individual participants spent no time fixating on a specific AOI. We modelled the average predicted number of saccades with a Gaussian LMM with population‐level effects group, emotion and their interaction. We predicted the total number of saccades by extrapolating the number made during the viewing period to the full length of the video. To explore the first fixations and the last fixations before stopping the video, we additionally constructed two categorical LMMs.

## RESULTS

### Self‐reported clinical assessments

Groups differed in self‐reported clinical assessments of ADHD, ASD and alexithymia (see Table 1). Specifically, all clinical groups scored higher than the comparison group on the RADS‐R, on the ASRS‐v1.1 and on the TAS‐20 (see Table 2). Participants with ADHD+ASD or ASD scored higher on the RADS‐R than participants with ADHD, while participants with ADHD+ASD or ADHD scored higher on the ASRS‐v1.1 than participants with ASD. ADHD+ASD participants neither differed from ASD participants on the RADS‐R nor from ADHD participants on the ASRS‐v1.1. Furthermore, clinical groups did not differ from each other on the TAS‐20, except for ASD participants scoring credibly higher than ADHD participants.

**TABLE 2:** Log‐transformed Bayes Factors for posthoc group comparisons of self‐reported clinical assessments.

Measurement	ADHD+ASD versus ADHD	ADHD+ASD versus COMP	ADHD+ASD versus ASD	ADHD versus COMP	ADHD versus ASD	COMP versus ASD
ASRS‐v1.1	−.821	17.476*	7.154*	10.887*	2.713*	3.153*
RADS‐R	6.455*	20.162*	−1.176	5.270*	7.000*	20.337*
TAS‐20	.577	9.371*	.661	2.785*	6.479*	21.337*

*Note*: Asterisk indicates at least moderate evidence in favour of a group difference, corresponding to a log‐transformed Bayes Factor larger than 3.

Abbreviations: ASRS, Adult ADHD Self‐Report Scale; RADS‐R, Ritvo Autism Diagnostic Scale—Revised; TAS, Toronto Alexithymia Scale.

### Emotion discrimination threshold

Mean inter‐item correlation of all 64 videos was .311, and Cronbach's alpha was .966. Item discrimination ranged from .178 to .733 (mean = .548 [.242, .708]), and item‐total correlation ranged from .261 to .722 (mean = .566 [.178, .733]).

We found credible differences between the adults with ADHD (COMP—ADHD: *estimate* = −.08 [−.15, −.02], *posterior probability* = 97.95%, *effect size* = .55 [.03, 1.07], see Figure 2) as well as between autistic adults and the comparison group (COMP—ASD: *estimate* = −.16 [−.23, −.09], *posterior probability* = 99.98%, *effect size* = 1.08 [.53, 1.61]). Although the estimates for the autistic sample decreased when including SDT in the model, effects between clinical groups and the comparison group remained credible (COMP—ADHD: *estimate* = −.09 [−.13, −.04], *posterior probability* = 99.99%, *effect size* = .66 [.34, .99]; COMP—ASD: *estimate* = −.1 [−.15, −.05], *posterior probability* = 100%, *effect size* = .77 [.40, 1.13]; COMP—ADHD+ASD: *estimate* = −.06 [−.1, −.02], *posterior probability* = 99.58%, *effect size* = .43 [.12, .74]). Differences between clinical groups were not credible (ASD—ADHD: *estimate* = .01 [−.03, .06], *posterior probability* = 70.79%, *effect size* = .11 [−.27, .49]; ASD—ADHD+ASD: *estimate* = .04 [0, .09], *posterior probability* = 96.17%, *effect size* = .33 [−.04, .70]; ADHD—ADHD+ASD: *estimate* = .03 [−.01, .07], *posterior probability* = 90.91%, *effect size* = .23 [−.10, .56]). However, equivalence tests between the clinical groups were undecided (inside ROPE: ADHD—ASD = 46.53%; ADHD+ASD—ASD = 12%; ADHD+ASD—ADHD = 27.75%). This model estimates that COMP adults need to watch 71.95% of the videos to correctly recognise the emotion, while autistic adults need to watch 81.86%, adults with ADHD 80.49% and adults with ADHD+ASD need to watch 77.54% of the videos. These inferences were unchanged when excluding outliers and including age as a covariate, but this model revealed a credible increase of EDT with age (see Supplementary Materials).

**FIGURE 2:**
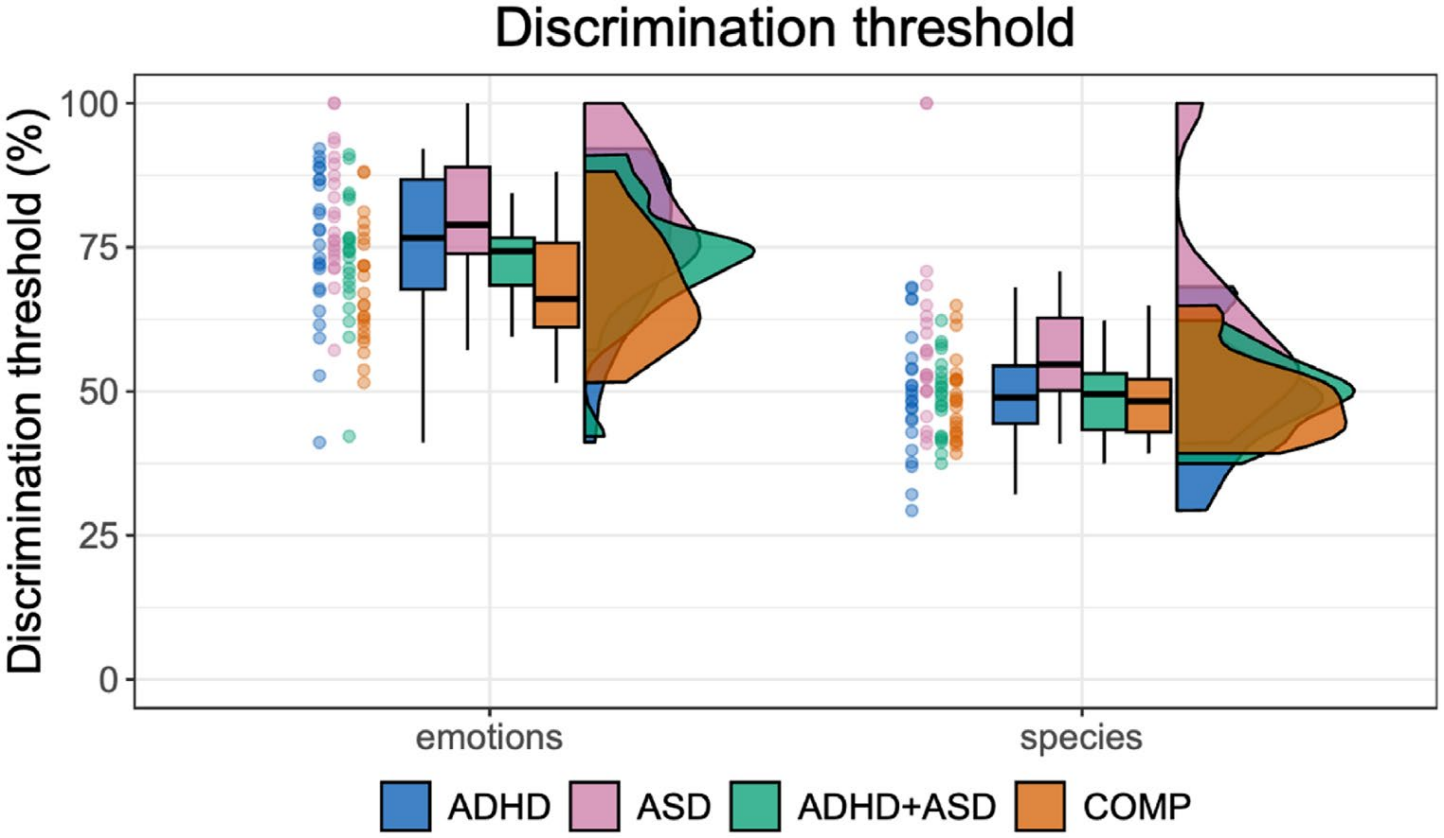
Visualisation of the discrimination thresholds in percent aggregated for emotions and species. Autistic participants and participants with ADHD exhibited increased discrimination thresholds for emotions associated with the facial expression. Their discrimination thresholds were comparable when recognising a species instead of an emotion.

These effects are independent of the emotion portrayed. Model comparison using the Bayes Factor revealed the model including emotion, group as well as SDT but not the interaction outperformed the other models, with extreme evidence in favour of this model compared to the model only including the intercept on the population‐level (log(*BF*) = 136.404; see Supplementary Materials S4.4). EDT was correlated with alexithymia as measured by the TAS‐20 only in the ADHD group (*r* = .42, *posterior probability* = 98.80%, for all other values see Supplementary Materials S4.5).

Last, the Bayesian ANOVA of the ranked SDT revealed only anecdotal evidence in favour of the model including the predictor group compared to the intercept‐only model (log*(BF)* = .211). Therefore, we can assume that the groups exhibited a similar discrimination threshold for recognising the species.

Accuracies were generally high, with a grand average of 87.99% accurate responses across groups in the FER task. The explorative analysis of the accuracy revealed no credible differences between any of the groups (see Supplementary Materials S5). However, accuracies were highest for happiness regardless of group (*estimate* = 1.06 [.63, 1.52], *posterior probability* = 100%).

### Gaze patterns

The model indicated no credible differences in dwell times between ADHD or ASD and COMP (COMP—ADHD: *estimate* = .09 [−.1, .28], *posterior probability* = 78.86%; COMP—ASD: *estimate* = .01 [−.18, .2], *posterior probability* = 53.49%), in contrast to our hypotheses (see Figures 3 and S6). Regardless of group, participants' dwell times were highest for the nose (*estimate* = .9 [.75, 1.05], *posterior probability* = 100%), followed by the eye region (*estimate* = .31 [.13, .48], *posterior probability* = 99.74%). Dwell times to specific AOIs also differed between emotions. Participants spent more time looking at the mouth when the facial expression was associated with happiness (*estimate* = .75 [.68, .82], *posterior probability* = 100%) or fear (*estimate* = .28 [.21, .34], *posterior probability* = 100%), and more time looking at the forehead when the facial expression was associated with anger (*estimate* = .24 [.17, .32], *posterior probability* = 100%).

**FIGURE 3:**
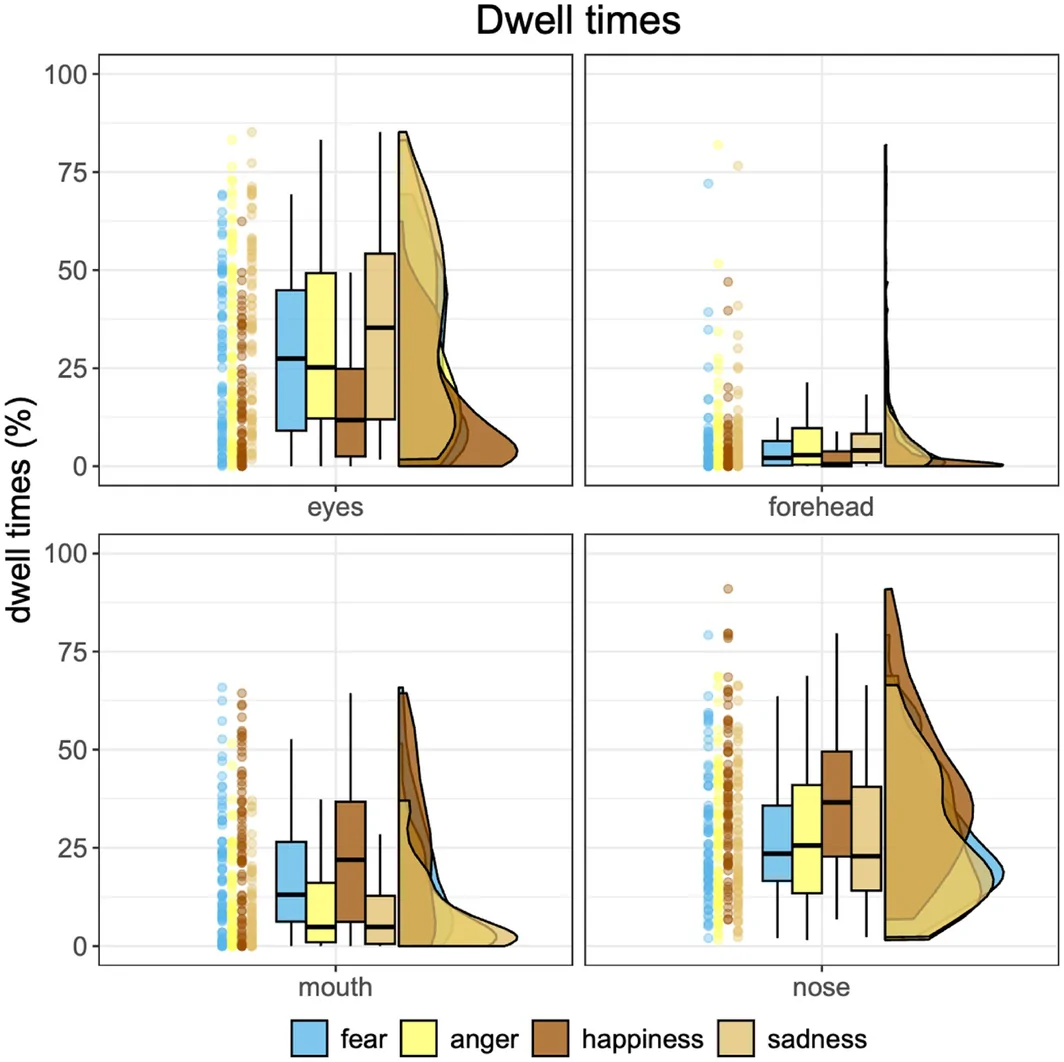
Dwell times for eyes, forehead, mouth and nose in the facial emotion recognition task as the percentage of total fixation duration. Dwell times seem to have differed between areas of interest (AOI), emotions and their interaction. Overall, participants dwelled more on the nose and less on the forehead, possibly due to the nose's centrality, which allows them to perceive the other AOIs in the periphery while focusing on the nose. Participants also dwelled more on the mouth when the emotion portrayed was either happiness or fear.

There were no credible differences between autistic and comparison participants in the number of saccades (COMP—ASD: *estimate* = −.1 [−.81, .63], *posterior probability* = 40.63%; see Supplementary Materials S6). However, the number differed between the emotions portrayed, with more saccades performed when a facial expression was associated with happiness (*estimate* = .44 [.31, .57], *posterior probability* = 100%) or fear (*estimate* = .31 [.18, .45], *posterior probability* = 100%), and fewer when it was associated with anger (*estimate* = −.55 [−.67, −.42], *posterior probability* = 100%; see Figure 4). Last, explorative models indicated no differences between groups with respect to the AOIs on which they fixated first or last. All participants were most likely to fixate on the nose or the eyes first. Similarly, last fixations were most likely placed on the nose or eye region, but there were some differences between emotions; for example, the mouth region was more likely if the facial expression was associated with happiness (see Supplementary Materials  S7).

**FIGURE 4:**
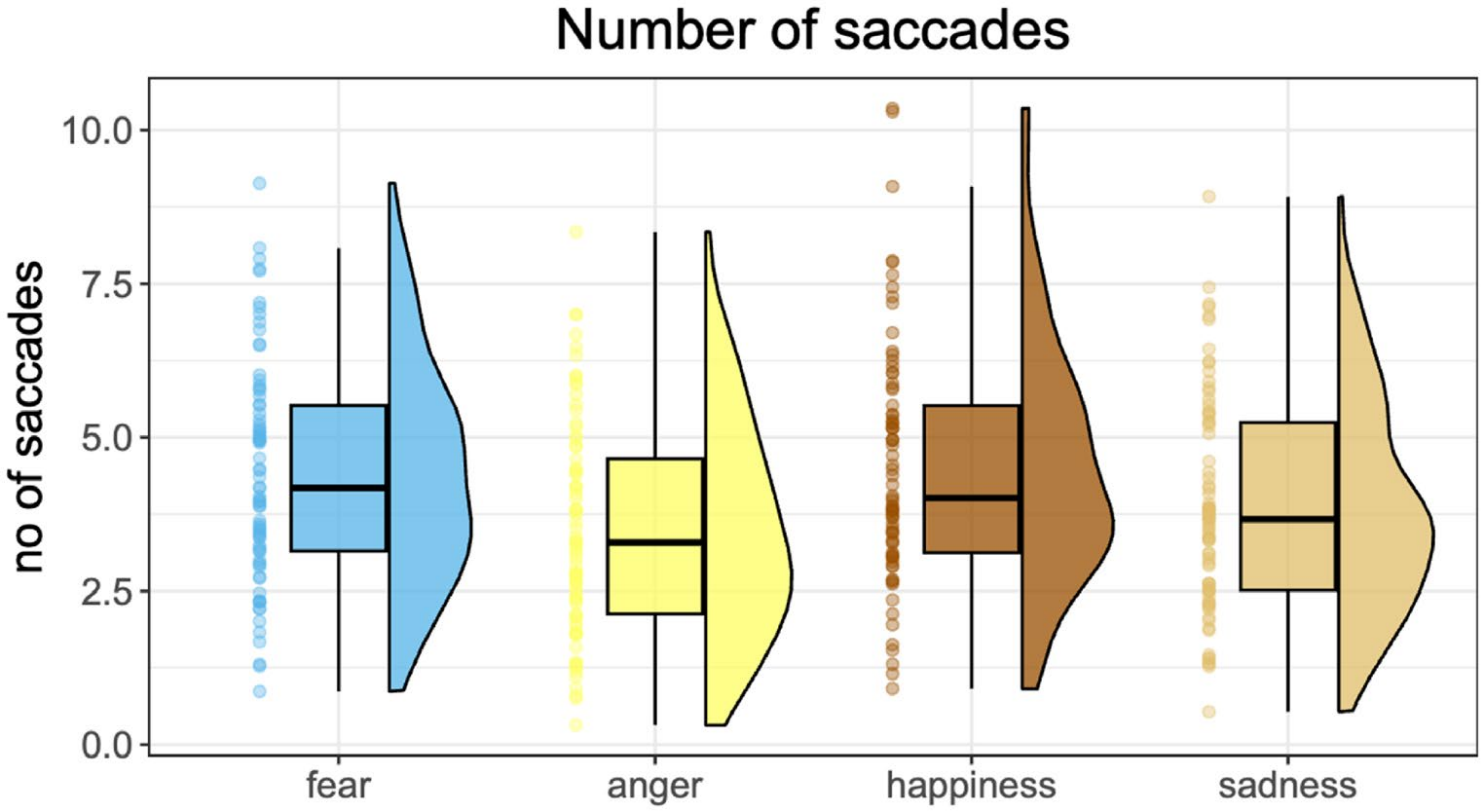
Number of saccades between AOIs. Since there were no credible differences between diagnostic groups, we plot differences between emotions, showing that anger seems to have triggered fewer saccades than the other emotions and sadness seems to have triggered fewer than fear and happiness.

## DISCUSSION

The current results provide evidence that both adults with ADHD and autistic adults exhibit specific difficulties recognising emotions in facial expressions compared to typically‐developing adults without any psychiatric diagnoses. These difficulties are independent of the emotion associated with the facial expression and manifest in increased discrimination thresholds for emotions but similar discrimination thresholds for species based on morphing tasks. Importantly, there was no credible difference in EDT between adults with ADHD and autistic adults, with similar but not equivalent estimates when taking the discrimination threshold of the control task into account. Furthermore, we explore these effects in a sample of adults with co‐occurring autism and ADHD and find similarly increased EDT compared to typically‐developing adults without any psychiatric diagnoses. Similarly, increased EDT in all three groups with autism and/or ADHD indicates that a co‐occurrence might not lead to exasperated differences with regards to FER.

In this study, autistic adults needed on average almost 16% more information to correctly recognise associated emotions compared to typically‐developing adults without any psychiatric diagnoses, while adults with ADHD needed about 8% more information in absolute terms. Including SDT, which draws on similar general processes of attention, face processing and motor reaction, decreased the difference in emotion discrimination threshold between autistic and non‐autistic adults, but the difference to typically‐developing adults remained credible, suggesting differences specific to emotion recognition. This model, including SDT, showed no credible differences between the emotion discrimination threshold of autistic adults with and without co‐occurring ADHD and adults with ADHD. Autistic adults with co‐occurring ADHD showed increased EDT to a similar degree as the autistic adults without co‐occurring ADHD and the adults with ADHD. The lack of an additive or interactive effect of the two conditions possibly suggests the same mechanism underlying attenuated EDT shared by autism and ADHD. The increased discrimination threshold for emotion in our groups of adults with autism and/or ADHD was not accompanied by differences in gaze patterns, suggesting differences at an interpretation level rather than a perceptual and information processing level.

These findings underscore the necessity of incorporating unbiased and specific FER measurements, like the EDT, into routine clinical assessments for both autism and ADHD, rather than viewing social‐cognitive deficits as exclusive to autism or secondary to inattention. A potential shared mechanism would suggest FER impairments as a transdiagnostic trait; thus, assessment protocols for ADHD should extend beyond executive function to include FER. Importantly, the observation that co‐occurrence of ADHD and ASD does not exacerbate FER deficits in individuals aged 17 to 45 years informs diagnostic expectations, suggesting that clinicians should not assume additive impairment or co‐occurrence. Furthermore, these findings indicate a specific FER impairment that is not explained by visual attention or general processes captured in the control task. Thus, therapeutic strategies should prioritise training emotion processing to improve EDT. Given the shared magnitude of impairment across groups when considering performance on the control task, unified social‐cognitive remediation programs may be effectively used across ASD, ADHD and co‐occurring ADHD+ASD without the need for differentiation.

Mirroring previous results (Calvo & Lundqvist, 2008), some emotions in our study were harder to recognise than others. Specifically, both EDT and accuracies indicate that happiness was the easiest to recognise, while fear and sadness were the hardest, with both results mirroring previous studies (Plank et al., 2022). However, the higher emotion discrimination threshold we observed in adults with ADHD as well as autistic adults with or without co‐occurring ADHD compared to typically‐developing adults without any psychiatric diagnoses was independent of the emotion participants attempted to recognise. Any differences between emotions were similar in all our groups. This general attenuation of emotion recognition is in line with a previous meta‐analysis on emotion recognition in autism (Yeung, 2022), while some studies suggest that fear may be more affected by ADHD than other emotions (Borhani & Nejati, 2018).

In our sample, we found a credible association between EDT and alexithymia measured with the TAS‐20 (Popp et al., 2008) in the ADHD group as well as a trend in the group with co‐occurring ADHD and ASD. While this might indicate an independent mechanism, readers should adopt a cautious interpretation of these findings. In our sample, the variance of TAS‐20 values in our autistic group was very narrow, with a standard deviation not only almost half of the standard deviation in the ADHD group in this study but also much narrower than previously reported variances in autistic samples (Kinnaird et al., 2019). This pattern might suggest that our sample did not capture the variance of alexithymia in ASD. Previous research also found no correlation between the TAS‐20 and a structured interview to assess alexithymia, which the authors argue is a more objective measure of alexithymia, in autistic and non‐autistic adults (Ryan et al., 2021). Furthermore, our analysis of the association between alexithymia and EDT was exploratory and should be assessed with confirmatory testing.

We did not observe any differences between adults with ADHD or autism and typically‐developing adults without any psychiatric diagnoses with regards to the accuracy with which they recognised the emotions. Since we designed the task for them to choose the amount of information they received themselves and overall accuracies were high, we conclude that all four groups were able to detect how much information they needed to correctly identify the emotion and rule out differences in strategies or awareness. Additionally, there was only anecdotal evidence in favour of differences between the groups in the control task, lending further support to our interpretation of discrimination threshold differences that are specific to emotion recognition.

In contrast to our expectations, we did not find any differences in gaze patterns between adults with ADHD or autism and typically‐developing adults without any psychiatric diagnoses. All four groups fixated the longest on the nose, which corresponds to the centre of the images, followed by the eye region. Percentage of fixation duration per AOI also differed between emotions with participants spending more time fixating on the mouth during videos where the facial expression was associated with happiness or fear. Previous literature on differences in fixation duration is mixed. While Kirchner et al. (2011) found no differences between autistic and non‐autistic adults, Reisinger et al. (2020) found a decrease in the number of fixations and proportion of fixation durations on the eyes in young autistic compared to non‐autistic people (age range from 3 to 25 years). Similarly, Bozkurt et al. (2024) found a decreased percentage of fixation duration in the eye region for children with ADHD compared to children without ADHD. These discrepancies between studies on children, adolescents and young adults and studies on adults could suggest that differences in gaze patterns decrease over time or that differences in paradigms contributed to the mixed results.

Our participants performed a comparable number of saccades and performed more saccades when observing a facial expression associated with fear or happiness as well as fewer when observing a facial expression associated with anger. In a previous study, non‐autistic adults produced more saccades between areas than autistic adults when observing an emotionally neutral facial expression during an identity recognition task (Yi et al., 2014). While emotionally neutral facial expressions may elicit greater differences in saccade behaviour, the difference in task – recognition of the emotion compared to familiarity – could also play an important role. For the assessment of familiarity, subtle differences between the facial features are important. In our task, we focused on emotions associated with distinct facial expressions. For example, only happiness is associated with raised cheeks and lips, which is reflected in increased fixation durations to the mouth region (Hamm et al., 2011). Therefore, producing fewer saccades may have been the better strategy, as indicated by our participants producing fewer than one saccade per second. Additionally, in a study employing a fast flickering task, no differences in saccades were observed (Vettori et al., 2020). Therefore, any possible differences in saccade behaviour during the observation of faces between autistic and non‐autistic adults could be dependent on the task.

We also explored whether our groups differed in the AOI on which they fixated first, before the associated emotion is detectable, and the AOI on which they fixated last before they decided that they have recognised the associated emotion. Again, we did not find any differences between adults with ADHD, adults with autism and typically‐developing adults without any psychiatric diagnoses. Our participants overwhelmingly fixated on the nose or the eyes first, probably because these are the areas closest to the fixation cross, which was presented before each video. They were also more likely to fixate on the eyes before stopping the video if the associated emotion was fear or the mouth if it was happiness. The fact that gaze patterns did not differ between the groups suggests that they focused their attention on the same AOIs, indicating similar information gathering strategies when recognising associated emotions in facial expressions. Possibly, these similar information gathering strategies indicate that differences in emotion discrimination threshold are caused by the interpretation of the information and not the sensory information itself.

When interpreting the results of this study, certain constraints on the generality should be considered (Simons et al., 2017). First, both our sample and stimuli consisted of mainly white Europeans. Therefore, it is unclear whether the findings generalise to different cultural contexts and ethnicities. Second, our sample only captured a part of the autistic population, namely, verbal autistic individuals without intellectual disability, restricting the generalisation of our findings to this subpopulation. Third, while we confirmed formal diagnoses based on existing diagnostic reports, we did not perform a clinical evaluation ourselves. Last, we added the sample of autistic adults with co‐occurring ADHD after preregistration and all comparisons with this sample should be interpreted carefully as they are exploratory.

## CONCLUSIONS

Both ASD and ADHD are associated with challenges in FER, a cornerstone of successful social interaction. Our study provides key insights into the nature of these challenges, showing specific difficulties with FER possibly stemming from shared mechanisms in ADHD and ASD, while general motor and sensory processing also used when interpreting the change from a human to an animal face were spared. Future research should prioritise identifying this possibly shared mechanism for a more unified understanding of social cognitive difficulties in both conditions. Furthermore, the findings are important for developing targeted interventions directly addressing the core social cognitive challenges faced by adults with ASD and ADHD, paving the way for more unified and effective strategies to support the social integration and well‐being of individuals with ADHD and ASD.

## AUTHOR CONTRIBUTIONS

**Irene Sophia Plank:** Conceptualization; investigation; funding acquisition; writing – original draft; methodology; validation; visualization; writing – review and editing; software; formal analysis; project administration; data curation; supervision; resources. **Kaltrina Krasniqi:** Investigation; writing – review and editing; project administration. **Alexandra Pior:** Investigation; project administration; writing – review and editing. **Julia Nowak:** Investigation; writing – review and editing; project administration. **Christine M. Falter‐Wagner:** Conceptualization; supervision; resources; writing – review and editing; funding acquisition.

## FUNDING INFORMATION

This project was funded by the FöFoLe of the Medical Faculty of the Ludwig Maximilian University of Munich through a starting grant received by ISP (reference number: 1161) and through funding for a dissertation project for KK (reference number: 19/2023). CFW was supported by German Research Council Funding (Deutsche Forschungsgemeinschaft, DFG, FA 876/3–1, FA 876/5–1). AP was supported by an Erasmus+ student mobility learning agreement for traineeships in higher education.

## CONFLICT OF INTEREST STATEMENT

The authors confirm that there are no conflicts of interest.

## Supporting information


Data S1.

## Data Availability

Materials, including data and code, have been made publicly available on OSF and can be accessed at https://doi.org/10.17605/OSF.IO/BM7TE. This study was preregistered prior to any data collection, including design, hypotheses and analysis plan (https://osf.io/jxacw).
