# Supplementary material for: No added cost: Emotion recognition in co‐occurring ADHD and ASD
Source: Br J Clin Psychol. 2026 Apr 12;65(4):981–99. doi: 10.1111/bjc.70056 (PMC13655947; doi:10.1111/bjc.70056)
Supplement: Supplementary file 1 [file BJC-65-981-s001.pdf]

Supplementary Materials

No added cost: emotion recognition in co-occurring ADHD and ASD

I. S. Plank

2025-12-19

Table of contents

1 Stimulus evaluation **1**
1.1 Reliability of the videos . . . 6

2 Model evaluation **7**

3 Plots **8**

4 Emotion discrimination threshold **10**
4.1 Model summary without SDT . . . 10
4.2 Model summary with SDT . . . 11
4.3 Comparison excluding outlier and including age . . . 12
4.4 Bayes Factor . . . 14
4.5 Correlation between EDT and TAS-20 . . . 15

5 Accuracy **15**
5.1 Model summary . . . 15
5.2 Bayes Factor . . . 17

6 Gaze patterns: FER **18**
6.1 Model summary for dwell times . . . 18
6.2 Model summary for saccades . . . 21

7 First and last fixation **22**
7.1 Model summary for first fixations . . . 22
7.2 Model summary for last fixations . . . 24

1 Stimulus evaluation

Video evaluation via SoSci

Irene Sophia Plank

2022-10-05

Setup

The data is loaded and relevant columns are selected. Then, it is transformed to the long format and the Video names are added. In the end, all information is contained in one data frame that is in the long format.

Choosing videos for the FER task

For the FER task, we want to choose the most natural video groups. Two things are important: that the videos are consistently rated as natural (median above 50) and that this is not due to a few very high ratings. First, outliers are removed that are lower than or equal to the 25% quantile minus 1.5 times the interquartile range. Then, face IDs are chosen who have the highest minima and a median per emotion of at least 50. We chose the median instead of the mean because the distributions are not at all normally distributed. The comments concerning the selected videos are saved to further improve them.

```
# get rid of extreme outliers for each video
df_fil = df %>% group_by(VID, FID, GEN) %>%
  filter(NAT > quantile(NAT, na.rm = T)[2] - 1.5*IQR(NAT, na.rm = T))

# aggregate per face ID and emotion
df_agg = df_fil %>% group_by(VID, GEN, EMO) %>%
  summarise(
    NAT_med = median(NAT, na.rm = T),
    NAT_min = min(NAT, na.rm = T),
    NAT_val = n()
  ) %>% filter(NAT_med >= 50 & NAT_val > 10)
```

```
## `summarise()` has grouped output by 'VID', 'GEN'. You can override using the
## `.groups` argument.
```

```
# choose the videos with the highest minima
n = 8 # number of videos per gender and emotion
vid_sel = df_agg %>%
  arrange(desc(NAT_min)) %>%
  group_by(GEN, EMO) %>%
  slice(1:n) %>%
  select(GEN, EMO, VID)

# filter the full data frame to only include selected videos
df_FER_sel = df_fil %>% filter(VID %in% vid_sel$VID)

# aggregate info for the selected videos
df_FER_sel_agg = df_FER_sel %>% group_by(FID, VID, EMO, GEN) %>%
  summarise(
    NAT_avg = mean(NAT, na.rm = T),
    NAT_ste = sd(NAT, na.rm = T) / sqrt(n()),
    NAT_med = median(NAT, na.rm = T),
    NAT_min = min(NAT, na.rm = T)
  ) %>%
  arrange(FID, EMO, VID) %>%
  mutate(
    EMO     = as.factor(EMO),
    GEN     = as.factor(GEN)
  )
```

```
## `summarise()` has grouped output by 'FID', 'VID', 'EMO'. You can override using
## the `.groups` argument.
```

```
kable(df_FER_sel_agg)
```

FID	VID	EMO	GEN	NAT_avg	NAT_ste	NAT_med	NAT_min
F01	AF01AFS_gimped	AF	F	65.48000	4.679430	65.0	30
F01	AF01ANS_gimped	AN	F	80.08696	4.076789	85.0	37

FID	VID	EMO	GEN	NAT_avg	NAT_ste	NAT_med	NAT_min
F01	AF01HAS_gimped	HA	F	70.14286	4.723440	74.0	27
F01	AF01SAS_gimped	SA	F	65.40000	5.216640	66.0	21
F02	AF02AFS_gimped	AF	F	78.96000	3.592344	81.0	41
F02	BF02HAS_gimped	HA	F	82.81250	4.041677	84.5	46
F02	AF02SAS_gimped	SA	F	60.78947	5.372977	65.0	27
F05	AF05AFS_gimped	AF	F	74.84000	4.412361	81.0	25
F05	AF05ANS_gimped	AN	F	65.60000	4.720876	63.0	21
F05	AF05HAS_gimped	HA	F	72.09091	3.921254	74.5	21
F05	AF05SAS_gimped	SA	F	69.69565	4.852950	72.0	21
F07	BF07ANS_gimped	AN	F	65.56000	4.519248	68.0	21
F07	BF07SAS_gimped	SA	F	64.12000	4.836776	61.0	25
F08	BF08HAS_gimped	HA	F	73.52381	3.566791	76.0	31
F09	AF09AFS_gimped	AF	F	74.92308	7.346254	83.0	23
F09	AF09HAS_gimped	HA	F	70.04000	5.441960	81.0	10
F09	AF09SAS_gimped	SA	F	79.50000	3.979112	86.0	38
F11	AF11SAS_gimped	SA	F	65.32000	5.179356	68.0	25
F14	AF14ANS_gimped	AN	F	63.52000	5.400654	63.0	26
F14	AF14SAS_gimped	SA	F	72.68000	4.248027	74.0	37
F16	AF16AFS_gimped	AF	F	63.76000	5.407489	75.0	26
F16	AF16HAS_gimped	HA	F	58.87500	5.785199	63.5	11
F19	AF19HAS_gimped	HA	F	63.40000	6.081940	73.0	10
F20	AF20AFS_gimped	AF	F	70.50000	5.208341	77.5	22
F20	AF20ANS_gimped	AN	F	71.36000	4.116989	69.0	30
F20	AF20SAS_gimped	SA	F	66.12000	4.294306	70.0	25
F24	AF24AFS_gimped	AF	F	61.04167	5.294747	60.0	25
F24	AF24ANS_gimped	AN	F	73.52000	4.004214	78.0	31
F29	AF29AFS_gimped	AF	F	68.04000	4.747448	69.0	28
F29	AF29ANS_gimped	AN	F	64.76000	5.172775	66.0	18
F29	AF29HAS_gimped	HA	F	66.24000	4.966313	75.0	17
F32	AF32ANS_gimped	AN	F	69.92000	4.535754	77.0	25
M01	AM01AFS_gimped	AF	M	73.40000	4.277481	72.5	34
M01	AM01ANS_gimped	AN	M	79.05882	3.203250	76.0	56
M01	AM01SAS_gimped	SA	M	67.27778	5.642281	71.5	21
M02	AM02ANS_gimped	AN	M	64.80952	5.251584	63.0	22
M02	AM02HAS_gimped	HA	M	76.05263	3.561879	75.0	55
M03	AM03AFS_gimped	AF	M	71.33333	4.703764	71.0	29
M03	AM03SAS_gimped	SA	M	69.75000	4.561207	68.0	30
M05	AM05AFS_gimped	AF	M	70.61905	4.419910	75.0	34
M05	AM05ANS_gimped	AN	M	59.80952	5.367281	56.0	16
M06	AM06SAS_gimped	SA	M	65.95238	5.105308	73.0	23
M07	AM07AFS_gimped	AF	M	66.38095	5.654108	69.0	21
M07	AM07SAS_gimped	SA	M	63.52381	4.332391	65.0	27
M09	AM09HAS_gimped	HA	M	85.35294	2.400890	84.0	64
M09	AM09SAS_gimped	SA	M	77.70000	3.935065	80.5	40

FID	VID	EMO	GEN	NAT_avg	NAT_ste	NAT_med	NAT_min
M10	AM10AFS_gimped	AF	M	74.76190	5.085415	86.0	36
M10	AM10HAS_gimped	HA	M	82.72222	3.545968	83.5	46
M11	AM11AFS_gimped	AF	M	74.47619	4.552142	78.0	33
M11	AM11ANS_gimped	AN	M	79.00000	3.277515	80.5	46
M11	AM11HAS_gimped	HA	M	79.94444	3.257442	79.0	60
M13	AM13AFS_gimped	AF	M	73.60000	4.660811	81.0	30
M13	AM13HAS_gimped	HA	M	79.42857	4.060009	82.0	31
M13	AM13SAS_gimped	SA	M	67.14286	5.033358	78.0	23
M14	AM14ANS_gimped	AN	M	76.85714	4.398747	79.0	35
M14	AM14HAS_gimped	HA	M	76.04762	4.027569	76.0	42
M14	AM14SAS_gimped	SA	M	72.57143	5.222466	74.0	21
M23	BM23ANS_gimped	AN	M	55.85714	6.508707	63.0	12
M23	BM23SAS_gimped	SA	M	57.28571	5.424435	63.0	19
M27	BM27HAS_gimped	HA	M	66.33333	4.939796	65.0	22
M28	BM28ANS_gimped	AN	M	69.25000	4.566396	73.5	25
M28	BM28HAS_gimped	HA	M	78.33333	3.699496	79.0	41
M31	BM31AFS_gimped	AF	M	73.60000	4.718608	73.5	32
M31	BM31ANS_gimped	AN	M	71.00000	5.366127	78.0	23

```
# # get all comments
# df_FER_sel_COM = df_FER_sel %>% filter(!is.na(COM)) %>% select(GEN, FID, VID, COM) %>%
#   arrange(FID, VID)
# kable(df_FER_sel_COM)

# drop all unnecessary variables
rm(list = setdiff(ls(), c("df", "df_FER_sel", "df_FER_sel_agg", "df_FER_sel_COM")))
```

Visualisation

```
# set colour scheme
cls = c("#0072B2", "#009E73")
names(cls) = c("M", "F")

# create boxplots
ggplot(df_FER_sel, aes(x = EMO, y = NAT, fill = GEN)) +
  geom_boxplot() +
  labs (x = "emotion", y = "natural") +
  ggtitle("Average natural rating") +
  theme_classic() + scale_fill_manual(values = cls) +
  theme(legend.position = "bottom", plot.title = element_text(hjust = 0.5))
```

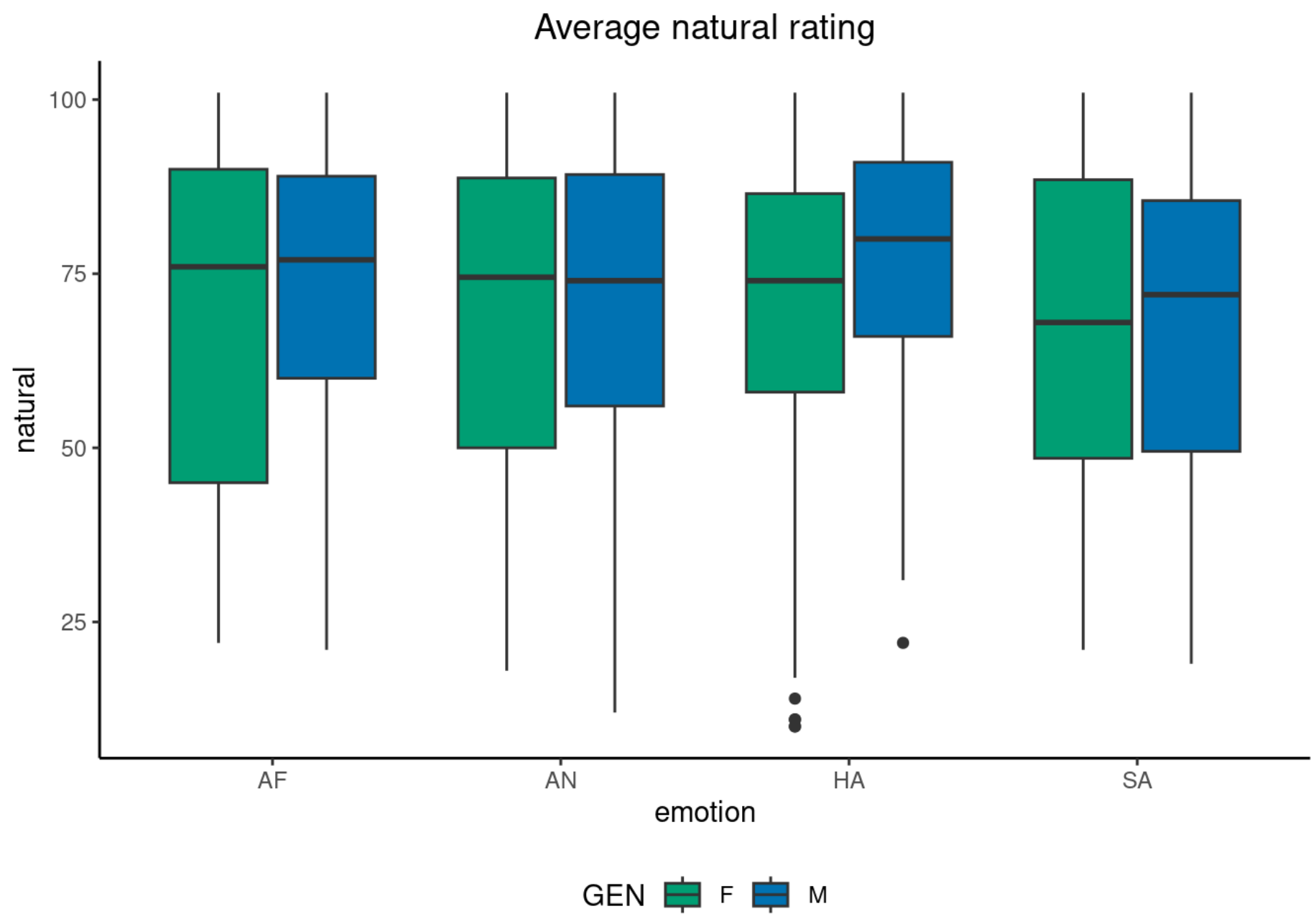

Gender and emotion effects

To stay in line with our main analysis, we compute a Bayesian ANOVA to assess possible differences in naturalness between emotions and gender expression.

```
# check normal distribution
kable(df_FER_sel_agg %>%
        group_by(EMO, GEN) %>%
        rstatix::shapiro_test(NAT_avg) %>%
        mutate(
sig = if_else(p < 0.05, "*", "")
        ))
```

EMO	GEN	variable	statistic	p	sig
AF	F	NAT_avg	0.9628623	0.8368830	
AF	M	NAT_avg	0.8284125	0.0571510	
AN	F	NAT_avg	0.8958537	0.2649964	
AN	M	NAT_avg	0.9221722	0.4477364	
HA	F	NAT_avg	0.9692711	0.8922278	
HA	M	NAT_avg	0.9127296	0.3736861	
SA	F	NAT_avg	0.9112234	0.3627591	
SA	M	NAT_avg	0.9806486	0.9660427	

```
# now we can compute our ANOVA
BayesFactor::anovaBF(NAT_avg ~ GEN * EMO, data = df_FER_sel_agg)
```

```
## Warning: data coerced from tibble to data frame
```

```
## Bayes factor analysis
## --------------
## [1] EMO                 : 1.079402  ±0%
## [2] GEN                 : 0.7966014 ±0.01%
## [3] EMO + GEN           : 0.9400547 ±1.06%
## [4] EMO + GEN + EMO:GEN : 0.5766506 ±1.16%
##
## Against denominator:
##   Intercept only
## ---
## Bayes factor type: BFlinearModel, JZS
```

1.1 Reliability of the videos

```
# Item Reliability

Item   | Alpha if deleted | Total Correlation | Discrimination
--------------------------------------------------------------
AM13HA |             0.97 |              0.62 |           0.60
AF29HA |             0.97 |              0.62 |           0.60
AM06SA |             0.97 |              0.50 |           0.48
AM09SA |             0.97 |              0.39 |           0.38
AF24AN |             0.97 |              0.62 |           0.60
AF05HA |             0.97 |              0.49 |           0.46
AM01AN |             0.97 |              0.70 |           0.68
AF05SA |             0.97 |              0.48 |           0.46
BF08HA |             0.97 |              0.43 |           0.41
AM01AF |             0.97 |              0.59 |           0.57
AM10AF |             0.97 |              0.56 |           0.54
BM27HA |             0.97 |              0.52 |           0.50
AF11SA |             0.97 |              0.60 |           0.59
AM05AF |             0.97 |              0.64 |           0.62
AF24AF |             0.97 |              0.37 |           0.35
BM23AN |             0.97 |              0.27 |           0.25
AM02HA |             0.97 |              0.52 |           0.49
AM07AF |             0.97 |              0.38 |           0.37
AM02AN |             0.97 |              0.64 |           0.62
BM28AN |             0.97 |              0.62 |           0.60
AF05AF |             0.97 |              0.18 |           0.17
AM11AF |             0.97 |              0.58 |           0.56
AF14AN |             0.96 |              0.73 |           0.71
AF01HA |             0.97 |              0.53 |           0.51
AM14HA |             0.97 |              0.60 |           0.57
AF16AF |             0.97 |              0.67 |           0.66
BF02HA |             0.97 |              0.64 |           0.62
AF20AN |             0.97 |              0.69 |           0.68
AF20AF |             0.97 |              0.68 |           0.67
AF29AF |             0.97 |              0.53 |           0.51
AM14SA |             0.96 |              0.73 |           0.72
AF05AN |             0.97 |              0.66 |           0.65
AM07SA |             0.97 |              0.45 |           0.43
BM31AF |             0.97 |              0.25 |           0.23
BM31AN |             0.97 |              0.57 |           0.55
AM13SA |             0.97 |              0.54 |           0.53
AF16HA |             0.97 |              0.61 |           0.59
AF29AN |             0.97 |              0.64 |           0.62
AM09HA |             0.97 |              0.45 |           0.42
BM28HA |             0.97 |              0.63 |           0.61
AF14SA |             0.97 |              0.62 |           0.60
AF01AN |             0.97 |              0.60 |           0.58
AF09AF |             0.97 |              0.42 |           0.41
BF07SA |             0.97 |              0.61 |           0.59
AF09HA |             0.97 |              0.70 |           0.68
BF07AN |             0.97 |              0.58 |           0.57
AF02AF |             0.97 |              0.54 |           0.52
AF09SA |             0.97 |              0.70 |           0.69
AM14AN |             0.97 |              0.64 |           0.62
AM11HA |             0.97 |              0.55 |           0.52
AM10HA |             0.97 |              0.70 |           0.69
AM01SA |             0.97 |              0.49 |           0.48
AM11AN |             0.97 |              0.55 |           0.53
AF20SA |             0.97 |              0.62 |           0.61
```

```
AM03AF |              0.97 |                0.53 |            0.52
AF01AF |              0.97 |                0.71 |            0.70
AF19HA |              0.97 |                0.47 |            0.45
AF32AN |              0.97 |                0.64 |            0.62
AF01SA |              0.97 |                0.72 |            0.70
BM23SA |              0.97 |                0.66 |            0.64
AM05AN |              0.97 |                0.67 |            0.66
AM03SA |              0.97 |                0.51 |            0.49
AF02SA |              0.97 |                0.57 |            0.55
AM13AF |              0.97 |                0.50 |            0.48

Mean inter-item-correlation = 0.311  Cronbach's alpha = 0.966
```

```
# A tibble: 3 x 7
  name                   mean       sd ci.low ci.high   min   max
  <chr>                 <dbl>    <dbl>  <dbl>   <dbl> <dbl> <dbl>
1 Alpha_if_deleted      0.966 0.000281  0.966   0.967 0.965 0.967
2 Discrimination        0.548 0.117     0.242   0.708 0.165 0.718
3 Item_Total_Correlation 0.566 0.117     0.261   0.722 0.178 0.733
```

2 Model evaluation

We evaluated data-naive models using simulation-based calibration to perform prior predictive checks and checks of computational faithfulness and model sensitivity. After running the models, we also performed posterior predictive checks to assess model fit before interpreting the model scores.

Due to a large variation in difficulty between the individual videos, posterior fit of the following models was suboptimal and aggregated values were modelled instead:

- emotion discrimination threshold
- species discrimination threshold
- fixation durations
- saccades

Full data was modelled for the following outcome variables:

- accuracies
- first fixation
- last fixation

More information including the output of all checks can be found on the OSF repository of the project: https://doi.org/10.17605/OSF.IO/BM7TE

3 Plots

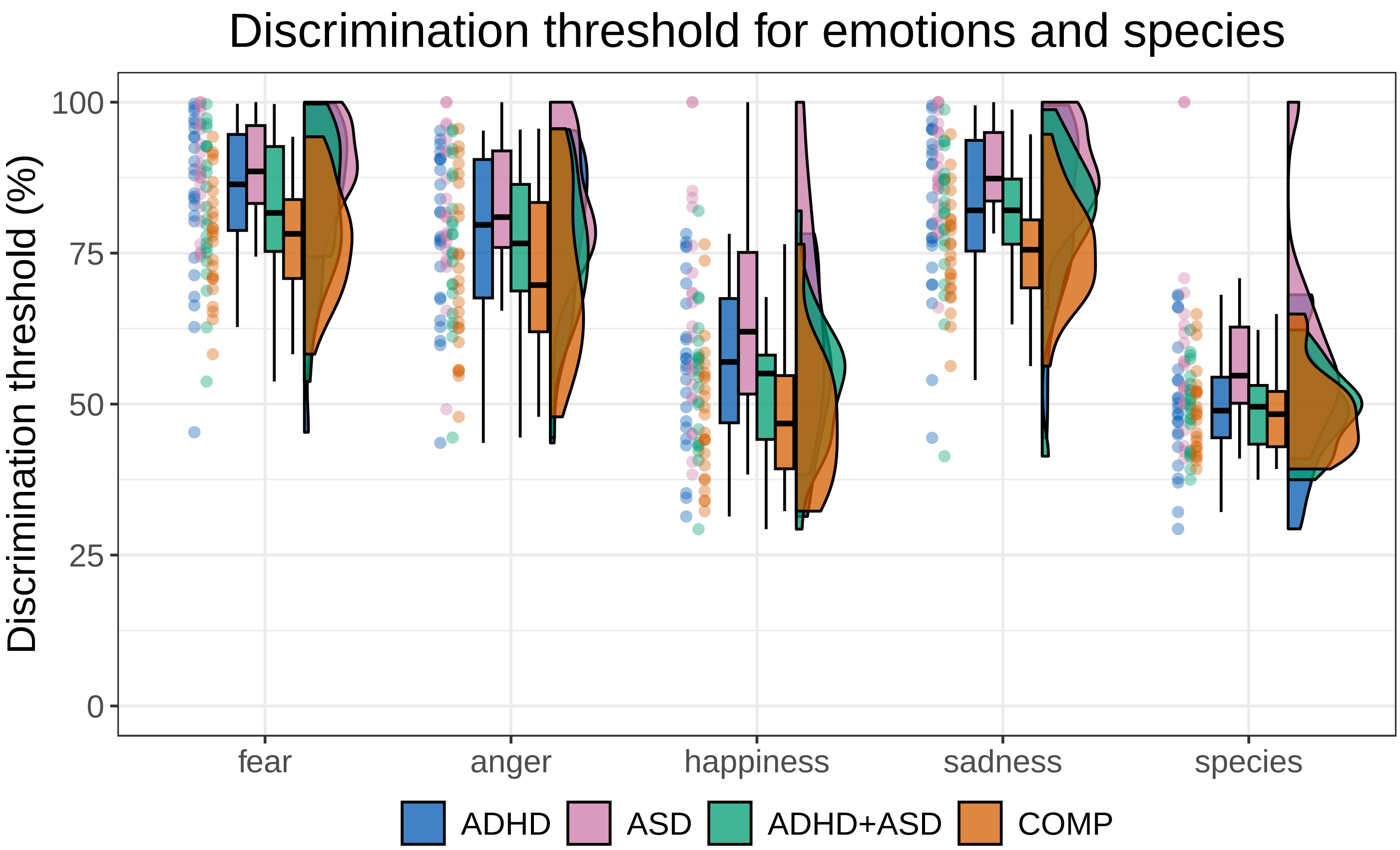
Discrimination threshold for emotions and species
Discrimination threshold (%)
100
75
50
25
0
fear
anger
happiness
sadness
species
ADHD
ASD
ADHD+ASD
COMP

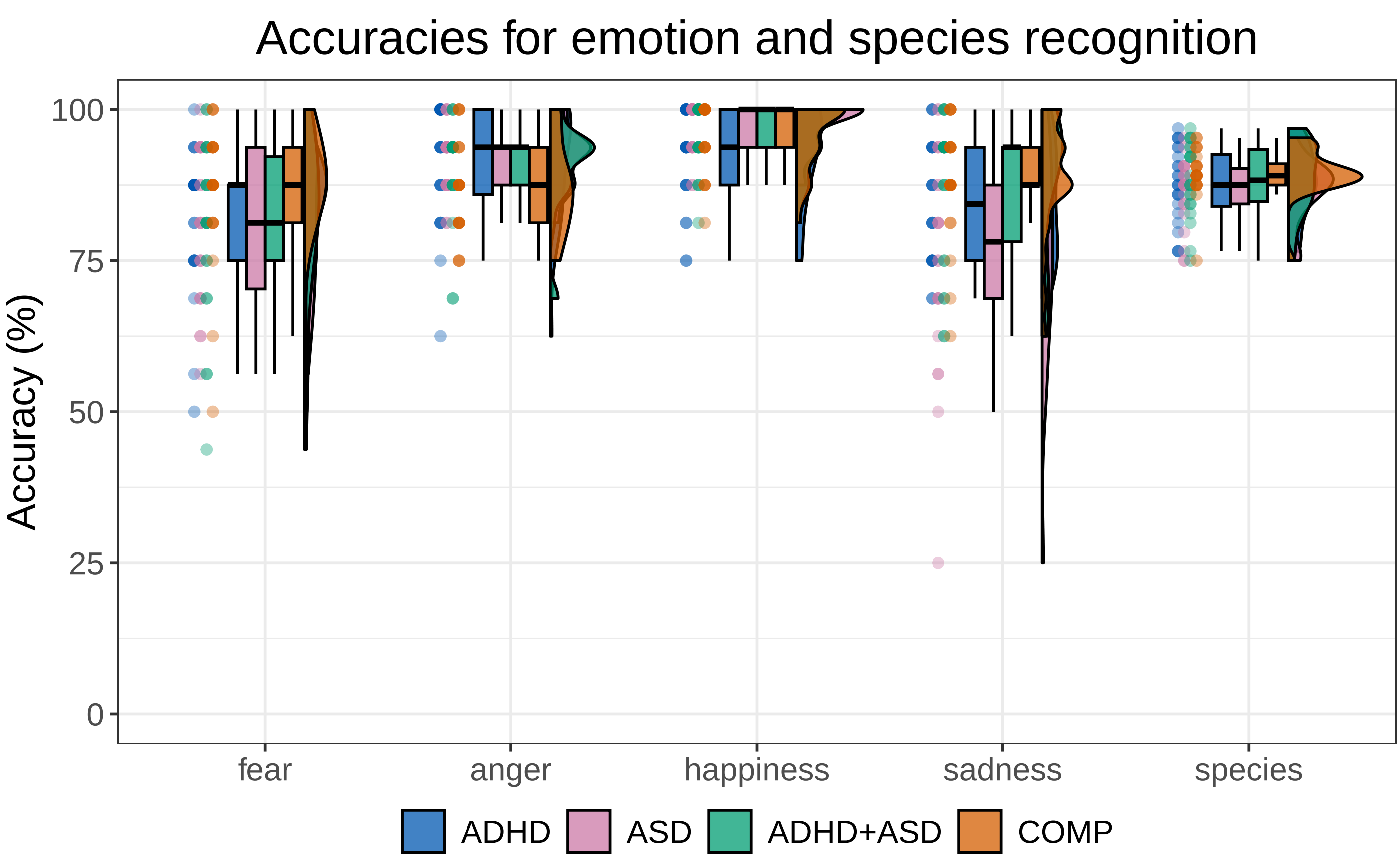
Accuracies for emotion and species recognition
Accuracy (%)
100
75
50
25
0
fear
anger
happiness
sadness
species
ADHD
ASD
ADHD+ASD
COMP

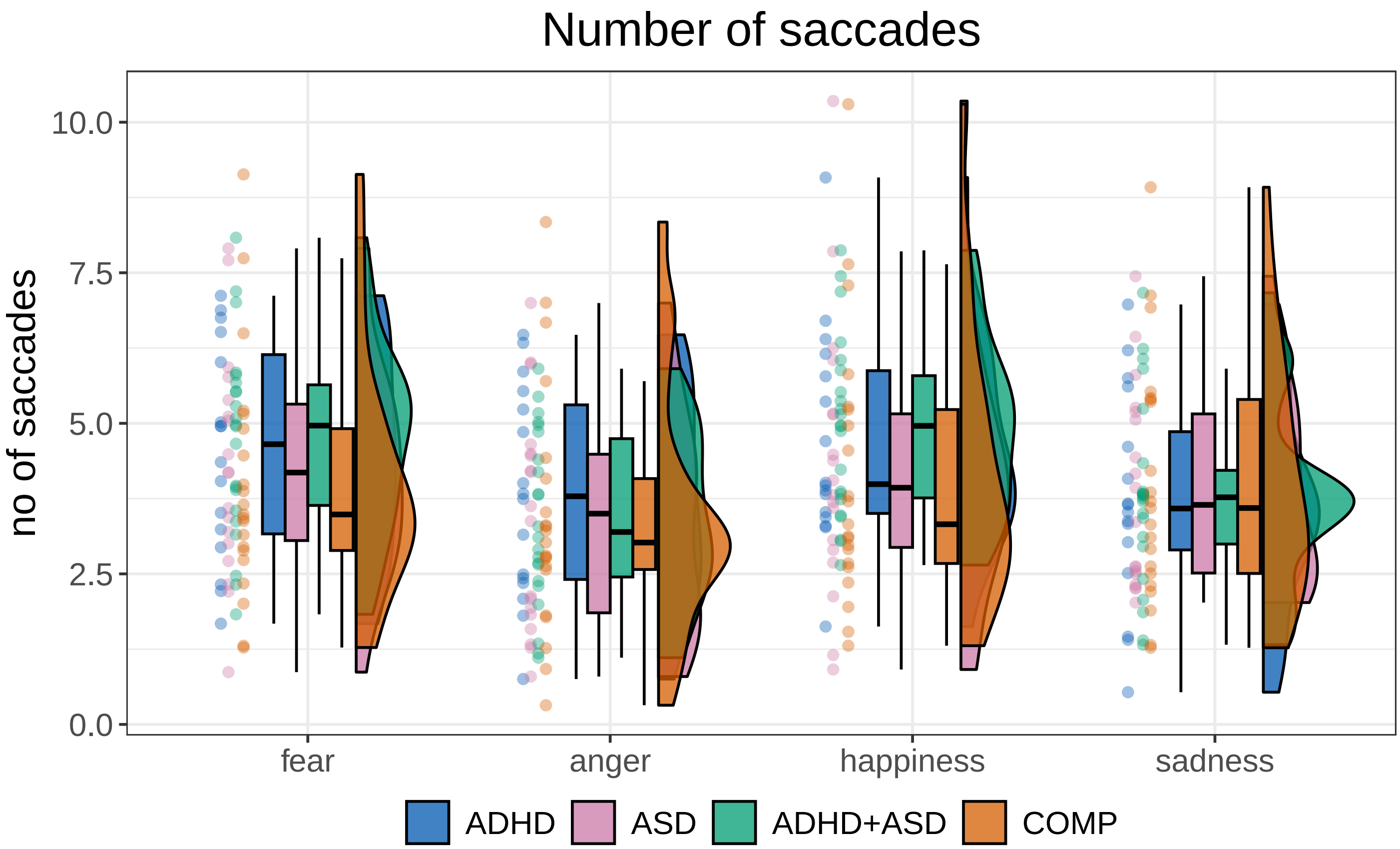
Number of saccades
no of saccades
10.0
7.5
5.0
2.5
0.0
fear
anger
happiness
sadness
ADHD
ASD
ADHD+ASD
COMP

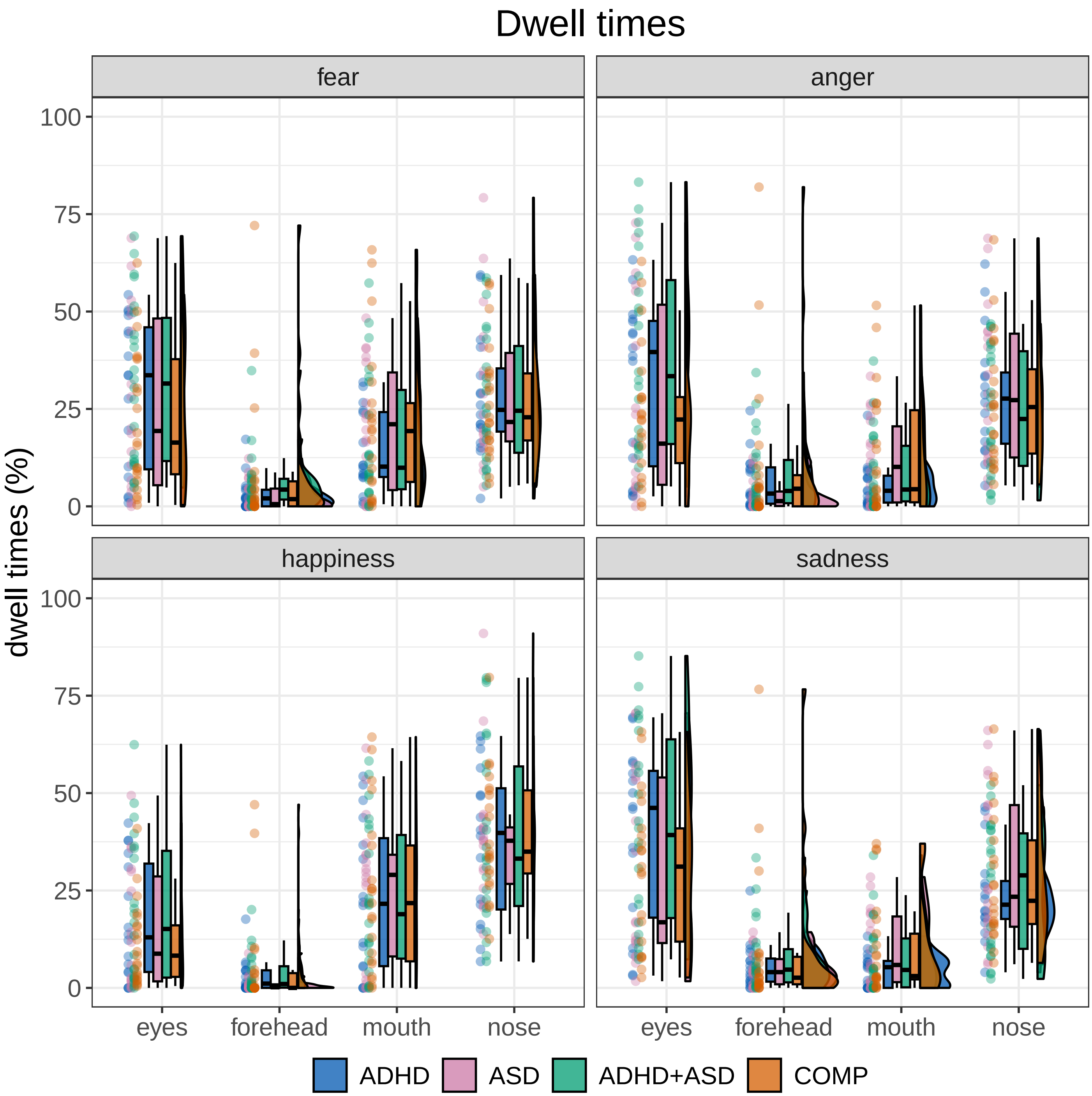

4 Emotion discrimination threshold

4.1 Model summary without SDT

```
[1] "Contrast settings for Emotion"


   [,1] [,2] [,3]
AF    1    0    0
AN    0    1    0
HA    0    0    1
SA   -1   -1   -1


[1] "Contrast settings for Diagnosis"
```

```
     [,1] [,2] [,3]
ADHD    1    0    0
ASD     0    1    0
COMP   -1   -1   -1
BOTH    0    0    1
```

```
 Family: gaussian
  Links: mu = identity; sigma = identity
Formula: disc | trunc(lb = 0, ub = 1) ~ diagnosis * emo + (1 | subID)
   Data: df.fer.agg (Number of observations: 368)
  Draws: 4 chains, each with iter = 3000; warmup = 1000; thin = 1;
         total post-warmup draws = 8000

Multilevel Hyperparameters:
~subID (Number of levels: 92)
              Estimate Est.Error l-95% CI u-95% CI Rhat Bulk_ESS Tail_ESS
sd(Intercept)     0.13      0.01     0.11     0.16 1.00     1654     3064

Regression Coefficients:
                Estimate Est.Error l-95% CI u-95% CI Rhat Bulk_ESS Tail_ESS
Intercept           0.76      0.01     0.73     0.78 1.00     1031     2027
diagnosis1          0.01      0.02    -0.04     0.06 1.00      976     1974
diagnosis2          0.08      0.02     0.04     0.13 1.00     1203     2244
diagnosis3         -0.02      0.02    -0.07     0.03 1.01      910     1999
emo1                0.10      0.01     0.08     0.11 1.00    10577     6194
emo2                0.02      0.01     0.01     0.04 1.00    12145     6621
emo3               -0.20      0.01    -0.21    -0.19 1.00    11245     6333
diagnosis1:emo1     0.00      0.01    -0.02     0.02 1.00     7379     6287
diagnosis2:emo1    -0.00      0.01    -0.02     0.02 1.00     7400     6576
diagnosis3:emo1     0.00      0.01    -0.02     0.02 1.00     8362     6446
diagnosis1:emo2     0.00      0.01    -0.02     0.02 1.00     7812     6617
diagnosis2:emo2    -0.02      0.01    -0.04     0.00 1.00     7765     6689
diagnosis3:emo2     0.01      0.01    -0.01     0.03 1.00     7899     6203
diagnosis1:emo3     0.00      0.01    -0.02     0.02 1.00     7926     6656
diagnosis2:emo3     0.00      0.01    -0.02     0.02 1.00     7354     6141
diagnosis3:emo3    -0.00      0.01    -0.02     0.02 1.00     8059     6570

Further Distributional Parameters:
      Estimate Est.Error l-95% CI u-95% CI Rhat Bulk_ESS Tail_ESS
sigma     0.06      0.00     0.06     0.07 1.00     6134     6258

Draws were sampled using sample(hmc). For each parameter, Bulk_ESS
and Tail_ESS are effective sample size measures, and Rhat is the potential
scale reduction factor on split chains (at convergence, Rhat = 1).
```

4.2 Model summary with SDT

```
[1] "Contrast settings for Emotion"
```

```
   [,1] [,2] [,3]
AF    1    0    0
AN    0    1    0
HA    0    0    1
SA   -1   -1   -1
```

```
[1] "Contrast settings for Diagnosis"
```

```
     [,1] [,2] [,3]
ADHD    1    0    0
ASD     0    1    0
COMP   -1   -1   -1
BOTH    0    0    1

 Family: gaussian
  Links: mu = identity; sigma = identity
Formula: disc | trunc(lb = 0, ub = 1) ~ diagnosis * emo + sfsr.disc
   Data: df.fer.agg (Number of observations: 368)
  Draws: 4 chains, each with iter = 3000; warmup = 1000; thin = 1;
         total post-warmup draws = 8000

Regression Coefficients:
                Estimate Est.Error l-95% CI u-95% CI Rhat Bulk_ESS Tail_ESS
Intercept           0.78      0.01     0.76     0.80 1.00     6802     5902
diagnosis1          0.03      0.01    -0.00     0.05 1.00    10430     6383
diagnosis2          0.04      0.02     0.01     0.07 1.00     8460     6052
diagnosis3         -0.00      0.01    -0.03     0.02 1.00    10877     6840
emo1                0.11      0.02     0.08     0.14 1.00     9353     6541
emo2                0.02      0.01    -0.01     0.05 1.00    11223     5960
emo3               -0.22      0.01    -0.24    -0.19 1.00     8227     5972
sfsr.disc           0.11      0.01     0.09     0.13 1.00     9925     5480
diagnosis1:emo1     0.01      0.02    -0.04     0.05 1.00     8779     6461
diagnosis2:emo1     0.01      0.03    -0.05     0.06 1.00     7265     6332
diagnosis3:emo1    -0.00      0.02    -0.05     0.04 1.00     7600     5899
diagnosis1:emo2     0.00      0.02    -0.04     0.05 1.00     9396     6523
diagnosis2:emo2    -0.02      0.02    -0.07     0.03 1.00     7685     6044
diagnosis3:emo2     0.01      0.02    -0.04     0.05 1.00     8885     6393
diagnosis1:emo3    -0.00      0.02    -0.04     0.04 1.00     8578     5695
diagnosis2:emo3    -0.01      0.02    -0.05     0.03 1.00     7522     6222
diagnosis3:emo3     0.00      0.02    -0.04     0.04 1.00     8631     6343

Further Distributional Parameters:
      Estimate Est.Error l-95% CI u-95% CI Rhat Bulk_ESS Tail_ESS
sigma     0.13      0.01     0.12     0.14 1.00     6107     6008

Draws were sampled using sample(hmc). For each parameter, Bulk_ESS
and Tail_ESS are effective sample size measures, and Rhat is the potential
scale reduction factor on split chains (at convergence, Rhat = 1).
```

4.3 Comparison excluding outlier and including age

There were two autistic participants who watched all videos to completion, possibly misunderstanding the task instructions. Based on reviewer suggestions, we reran the model including SDT after excluding these two participants and adding age as a covariate.

```
[1] "Comparison of the estimates"
```

	disc | trunc(lb = 0, ub = 1)		disc | trunc(lb = 0, ub = 1)	
Predictors	Estimates	CI (95%)	Estimates	CI (95%)
Intercept	0.78	0.76 – 0.80	0.66	0.59 – 0.72
diagnosis1	0.02	-0.00 – 0.05	0.03	0.00 – 0.06
diagnosis2	0.04	0.01 – 0.07	0.03	0.00 – 0.06
diagnosis3	-0.00	-0.03 – 0.02	-0.01	-0.04 – 0.02
emo1	0.11	0.08 – 0.14	0.11	0.08 – 0.14

emo2	0.02	-0.01 – 0.05	0.02	-0.01 – 0.05
emo3	-0.22	-0.24 – -0.19	-0.21	-0.24 – -0.19
sfsr.disc	0.11	0.09 – 0.13	0.08	0.06 – 0.11
diagnosis1:emo1	0.01	-0.04 – 0.05	0.00	-0.04 – 0.05
diagnosis2:emo1	0.00	-0.05 – 0.06	0.01	-0.05 – 0.06
diagnosis3:emo1	-0.00	-0.05 – 0.04	-0.00	-0.05 – 0.05
diagnosis1:emo2	0.00	-0.04 – 0.05	0.00	-0.04 – 0.05
diagnosis2:emo2	-0.02	-0.07 – 0.03	-0.02	-0.07 – 0.03
diagnosis3:emo2	0.01	-0.04 – 0.05	0.01	-0.04 – 0.05
diagnosis1:emo3	-0.00	-0.04 – 0.04	0.00	-0.04 – 0.04
diagnosis2:emo3	-0.01	-0.05 – 0.03	-0.01	-0.05 – 0.04
diagnosis3:emo3	0.00	-0.04 – 0.04	-0.00	-0.04 – 0.04
age			0.00	0.00 – 0.01
Observations	368		359	
R^2 Bayes	0.612		0.605	

```
[1] "rerun H1a: COMP < ADHD"

Hypothesis Tests for class b:
                Hypothesis Estimate Est.Error CI.Lower CI.Upper Evid.Ratio
1 (0)-(2*diagnosis1... < 0    -0.09      0.02    -0.13    -0.05       7999
  Post.Prob Star
1         1    *
---
'CI': 95%-CI for one-sided and 97.5%-CI for two-sided hypotheses.
'*': For one-sided hypotheses, the posterior probability exceeds 97.5%;
for two-sided hypotheses, the value tested against lies outside the 97.5%-CI.
Posterior probabilities of point hypotheses assume equal prior probabilities.

[1] "rerun H1b: COMP < ASD"

Hypothesis Tests for class b:
                Hypothesis Estimate Est.Error CI.Lower CI.Upper Evid.Ratio
1 (0)-(2*diagnosis2... < 0    -0.09      0.02    -0.13    -0.04        Inf
  Post.Prob Star
1         1    *
---
'CI': 95%-CI for one-sided and 97.5%-CI for two-sided hypotheses.
'*': For one-sided hypotheses, the posterior probability exceeds 97.5%;
for two-sided hypotheses, the value tested against lies outside the 97.5%-CI.
Posterior probabilities of point hypotheses assume equal prior probabilities.

[1] "explore: ADHD < ASD"

Hypothesis Tests for class b:
                Hypothesis Estimate Est.Error CI.Lower CI.Upper Evid.Ratio
1 (diagnosis1)-(dia... < 0        0      0.02    -0.05     0.05       1.16
  Post.Prob Star
1      0.54
---
'CI': 95%-CI for one-sided and 97.5%-CI for two-sided hypotheses.
'*': For one-sided hypotheses, the posterior probability exceeds 97.5%;
for two-sided hypotheses, the value tested against lies outside the 97.5%-CI.
Posterior probabilities of point hypotheses assume equal prior probabilities.

[1] "explore: COMP < BOTH"
```

```
Hypothesis Tests for class b:
                Hypothesis Estimate Est.Error CI.Lower CI.Upper Evid.Ratio
1 (0)-(2*diagnosis3... < 0    -0.04      0.02    -0.09        0      55.74
  Post.Prob Star
1      0.98    *
---
'CI': 95%-CI for one-sided and 97.5%-CI for two-sided hypotheses.
'*': For one-sided hypotheses, the posterior probability exceeds 97.5%;
for two-sided hypotheses, the value tested against lies outside the 97.5%-CI.
Posterior probabilities of point hypotheses assume equal prior probabilities.

[1] "explore differences between diagnostic groups"

# Test for Practical Equivalence

  ROPE: [-0.02 0.02]

Parameter |        H0 | inside ROPE |           95% HDI
-------------------------------------------------------
ADHD-ASD  | Undecided |     53.79 % |     [-0.05, 0.05]
BOTH-ASD  | Undecided |     10.04 % | [-0.09, 2.14e-03]
BOTH-ADHD | Undecided |      9.82 % | [-0.08, 1.49e-03]

[1] "explore effect of facial species recognition task"

Hypothesis Tests for class b:
           Hypothesis Estimate Est.Error CI.Lower CI.Upper Evid.Ratio Post.Prob
1 (0)-(sfsr.disc) < 0    -0.08      0.01    -0.11    -0.06        Inf         1
  Star
1    *
---
'CI': 95%-CI for one-sided and 97.5%-CI for two-sided hypotheses.
'*': For one-sided hypotheses, the posterior probability exceeds 97.5%;
for two-sided hypotheses, the value tested against lies outside the 97.5%-CI.
Posterior probabilities of point hypotheses assume equal prior probabilities.

[1] "explore effect of age"

Hypothesis Tests for class b:
  Hypothesis Estimate Est.Error CI.Lower CI.Upper Evid.Ratio Post.Prob Star
1  (age) > 0        0         0        0     0.01       3999         1    *
---
'CI': 95%-CI for one-sided and 97.5%-CI for two-sided hypotheses.
'*': For one-sided hypotheses, the posterior probability exceeds 97.5%;
for two-sided hypotheses, the value tested against lies outside the 97.5%-CI.
Posterior probabilities of point hypotheses assume equal prior probabilities.
```

4.4 Bayes Factor

Table 2: Model comparison.

population-level	bf.log
diagnosis + emo + sfsr.disc	136.4040
emo + sfsr.disc	131.0742
diagnosis * emo + sfsr.disc	122.3387
diagnosis + emo	85.0294

Table 2: Model comparison.

population-level	bf.log
diagnosis + emo + diagnosis:emo	73.3344
emo	69.5555
diagnosis + sfsr.disc	32.8442
sfsr.disc	31.4413
diagnosis	7.3716

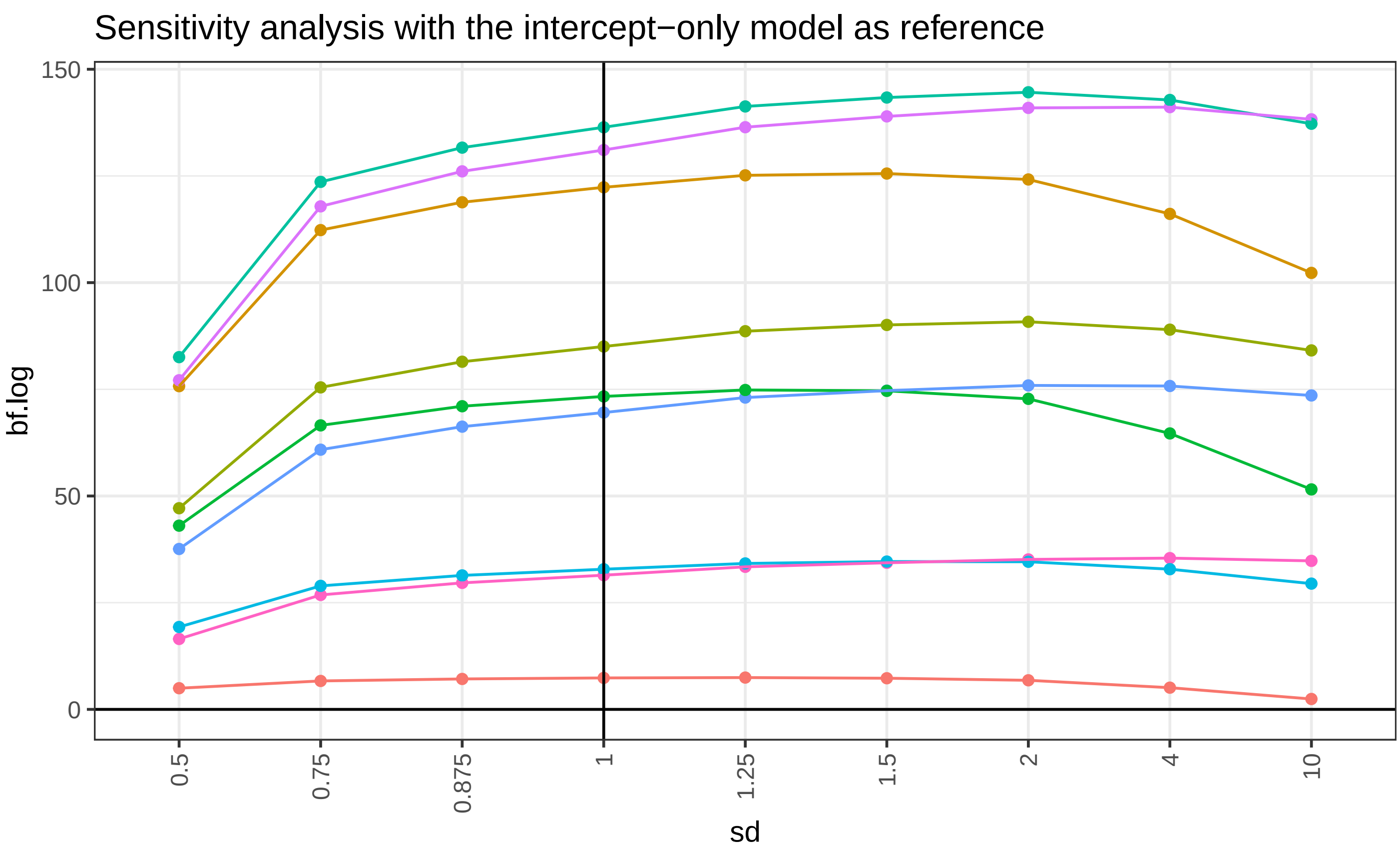

4.5 Correlation between EDT and TAS-20

Group	rho	CI_low	CI_high	pd	ROPE_Percentage	BF
ADHD	0.4070104	0.0758708	0.6729660	0.99000	0.03200	6.0165596
ASD	0.1035952	-0.2757547	0.4478253	0.70825	0.34875	0.5317468
COMP	0.1468017	-0.2048028	0.4990464	0.79350	0.30125	0.6001048
BOTH	0.3205353	-0.0493984	0.6272663	0.94375	0.10475	2.0080486

5 Accuracy

5.1 Model summary

```
[1] "Contrast settings for Emotion"

   [,1] [,2] [,3]
AF    1    0    0
AN    0    1    0
HA    0    0    1
SA   -1   -1   -1
```

```
[1] "Contrast settings for Diagnosis"

     [,1] [,2] [,3]
ADHD    1    0    0
ASD     0    1    0
COMP   -1   -1   -1
BOTH    0    0    1

 Family: bernoulli
  Links: mu = logit
Formula: acc.code ~ diagnosis * emo + (emo | subID) + (diagnosis | video)
   Data: df.fer (Number of observations: 5888)
  Draws: 4 chains, each with iter = 4000; warmup = 2000; thin = 1;
         total post-warmup draws = 8000

Multilevel Hyperparameters:
~subID (Number of levels: 92)
                    Estimate Est.Error l-95% CI u-95% CI Rhat Bulk_ESS Tail_ESS
sd(Intercept)           0.45      0.08     0.30     0.60 1.00     3747     5214
sd(emo1)                0.58      0.12     0.35     0.82 1.00     2674     3656
sd(emo2)                0.35      0.15     0.06     0.63 1.00     2129     2084
sd(emo3)                0.70      0.17     0.37     1.04 1.00     1339     3048
cor(Intercept,emo1)     0.13      0.23    -0.31     0.58 1.00     2383     3599
cor(Intercept,emo2)    -0.17      0.30    -0.71     0.45 1.00     4581     4729
cor(emo1,emo2)         -0.30      0.29    -0.77     0.36 1.00     4422     5316
cor(Intercept,emo3)    -0.02      0.25    -0.51     0.43 1.00     2607     4446
cor(emo1,emo3)         -0.17      0.25    -0.60     0.38 1.00     2063     3360
cor(emo2,emo3)          0.11      0.32    -0.51     0.72 1.00      915     1908

~video (Number of levels: 64)
                           Estimate Est.Error l-95% CI u-95% CI Rhat Bulk_ESS
sd(Intercept)                  0.89      0.10     0.72     1.11 1.00     2360
sd(diagnosis1)                 0.23      0.11     0.02     0.44 1.00     2930
sd(diagnosis2)                 0.20      0.11     0.01     0.43 1.00     2699
sd(diagnosis3)                 0.22      0.12     0.02     0.48 1.00     2269
cor(Intercept,diagnosis1)     -0.47      0.27    -0.88     0.17 1.00     5427
cor(Intercept,diagnosis2)      0.41      0.30    -0.32     0.85 1.00     6079
cor(diagnosis1,diagnosis2)    -0.28      0.36    -0.84     0.50 1.00     5837
cor(Intercept,diagnosis3)      0.21      0.30    -0.45     0.72 1.00     8120
cor(diagnosis1,diagnosis3)    -0.19      0.35    -0.78     0.54 1.00     5423
cor(diagnosis2,diagnosis3)     0.02      0.36    -0.66     0.70 1.00     5476
                           Tail_ESS
sd(Intercept)                  4096
sd(diagnosis1)                 2323
sd(diagnosis2)                 3027
sd(diagnosis3)                 3375
cor(Intercept,diagnosis1)      4447
cor(Intercept,diagnosis2)      4892
cor(diagnosis1,diagnosis2)     5888
cor(Intercept,diagnosis3)      6126
cor(diagnosis1,diagnosis3)     5942
cor(diagnosis2,diagnosis3)     6443

Regression Coefficients:
                Estimate Est.Error l-95% CI u-95% CI Rhat Bulk_ESS Tail_ESS
Intercept           2.59      0.14     2.33     2.87 1.00     2513     3760
diagnosis1         -0.21      0.12    -0.45     0.03 1.00     4648     5560
diagnosis2          0.02      0.14    -0.24     0.30 1.00     5095     5756
diagnosis3          0.08      0.13    -0.18     0.34 1.00     4621     5863
emo1               -0.54      0.20    -0.94    -0.15 1.00     2342     4112
```

```
emo2               -0.09      0.20    -0.47      0.31 1.00      2574      4037
emo3                1.06      0.22     0.63      1.52 1.00      3275      4941
diagnosis1:emo1     0.07      0.17    -0.26      0.41 1.00      5169      5302
diagnosis2:emo1    -0.13      0.18    -0.48      0.23 1.00      5354      5414
diagnosis3:emo1    -0.19      0.18    -0.54      0.17 1.00      5887      6261
diagnosis1:emo2     0.25      0.16    -0.07      0.57 1.00      6541      5431
diagnosis2:emo2     0.25      0.18    -0.09      0.60 1.00      6710      5972
diagnosis3:emo2    -0.03      0.17    -0.37      0.31 1.00      7172      5852
diagnosis1:emo3    -0.44      0.21    -0.86     -0.03 1.00      5881      4976
diagnosis2:emo3     0.58      0.26     0.08      1.09 1.00      5863      5376
diagnosis3:emo3    -0.02      0.24    -0.48      0.46 1.00      5752      5479

Draws were sampled using sample(hmc). For each parameter, Bulk_ESS
and Tail_ESS are effective sample size measures, and Rhat is the potential
scale reduction factor on split chains (at convergence, Rhat = 1).
```

5.2 Bayes Factor

Table 4: Model comparison.

population-level	bf.log
emo	2.4656
diagnosis + emo	-2.9377
diagnosis	-5.2980
diagnosis * emo	-7.8650

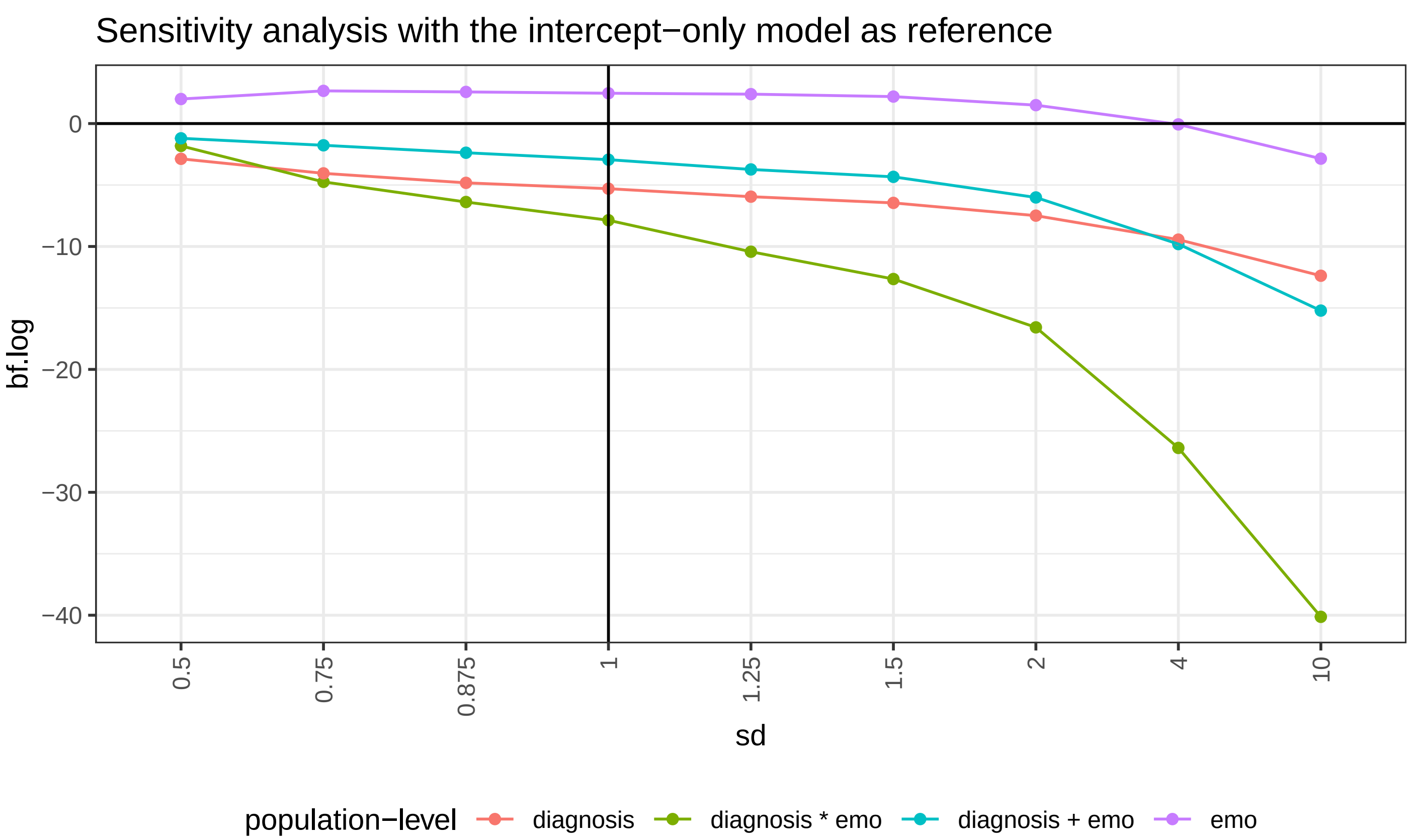

6 Gaze patterns: FER

6.1 Model summary for dwell times

```
[1] "Contrast settings for Emotion"

   [,1] [,2] [,3]
AF    1    0    0
AN    0    1    0
HA    0    0    1
SA   -1   -1   -1

[1] "Contrast settings for Diagnosis"

     [,1] [,2] [,3]
ADHD    1    0    0
ASD     0    1    0
BOTH    0    0    1
COMP   -1   -1   -1

[1] "Contrast settings for AOIs"

      [,1] [,2] [,3]
eyes     1    0    0
fore     0    1    0
mouth    0    0    1
nose    -1   -1   -1

 Family: hurdle_lognormal
  Links: mu = identity; sigma = identity; hu = identity
Formula: fix.perc ~ diagnosis * emo * AOI + (emo + AOI | subID)
   Data: df.fix (Number of observations: 1232)
  Draws: 4 chains, each with iter = 3000; warmup = 1000; thin = 1;
         total post-warmup draws = 8000

Multilevel Hyperparameters:
~subID (Number of levels: 77)
                    Estimate Est.Error l-95% CI u-95% CI Rhat Bulk_ESS Tail_ESS
sd(Intercept)           0.34      0.04     0.28     0.43 1.00     2521     4058
sd(emo1)                0.03      0.02     0.00     0.08 1.00     2865     4125
sd(emo2)                0.08      0.03     0.01     0.15 1.00     1492     1493
sd(emo3)                0.19      0.03     0.13     0.26 1.00     2117     3818
sd(AOI1)                1.01      0.08     0.86     1.19 1.00     3883     5758
sd(AOI2)                1.00      0.09     0.84     1.20 1.00     3803     5072
sd(AOI3)                1.14      0.09     0.99     1.33 1.00     3823     5471
cor(Intercept,emo1)     0.03      0.30    -0.57     0.60 1.00    15948     5851
cor(Intercept,emo2)     0.20      0.24    -0.31     0.63 1.00     7997     5740
cor(emo1,emo2)         -0.12      0.31    -0.69     0.51 1.00     1769     3743
cor(Intercept,emo3)    -0.07      0.16    -0.39     0.24 1.00     6215     6413
cor(emo1,emo3)          0.09      0.30    -0.52     0.66 1.01      703     1027
cor(emo2,emo3)         -0.59      0.22    -0.88    -0.04 1.01     1059     1224
cor(Intercept,AOI1)    -0.07      0.12    -0.31     0.18 1.00     3243     5218
cor(emo1,AOI1)          0.13      0.29    -0.48     0.65 1.00      517      729
cor(emo2,AOI1)         -0.38      0.23    -0.76     0.16 1.00      600      880
cor(emo3,AOI1)          0.65      0.11     0.40     0.84 1.00     2254     4647
cor(Intercept,AOI2)     0.35      0.11     0.11     0.56 1.00     3438     4886
cor(emo1,AOI2)          0.01      0.31    -0.60     0.60 1.01      498     1166
cor(emo2,AOI2)         -0.14      0.24    -0.58     0.34 1.00      914     1657
```

```
cor(emo3,AOI2)           0.31      0.15     0.01      0.58 1.00     2364     4518
cor(AOI1,AOI2)          -0.16      0.11    -0.38      0.06 1.00     4101     5794
cor(Intercept,AOI3)      0.04      0.12    -0.20      0.26 1.00     3795     5382
cor(emo1,AOI3)          -0.11      0.30    -0.65      0.52 1.02      350      886
cor(emo2,AOI3)           0.36      0.22    -0.15      0.74 1.00      823     1381
cor(emo3,AOI3)          -0.64      0.11    -0.83     -0.40 1.00     1159     2775
cor(AOI1,AOI3)          -0.44      0.09    -0.61     -0.25 1.00     4629     5728
cor(AOI2,AOI3)          -0.62      0.08    -0.75     -0.46 1.00     4136     5759

Regression Coefficients:
                     Estimate Est.Error l-95% CI u-95% CI Rhat Bulk_ESS
Intercept               -2.28      0.04    -2.37    -2.19 1.00     1899
diagnosis1               0.01      0.07    -0.12     0.15 1.00     2671
diagnosis2              -0.07      0.07    -0.21     0.06 1.00     2773
diagnosis3               0.04      0.06    -0.08     0.17 1.00     2417
emo1                     0.08      0.03     0.03     0.13 1.00     9255
emo2                    -0.03      0.03    -0.09     0.02 1.00     6192
emo3                    -0.08      0.03    -0.14    -0.01 1.00     5535
AOI1                     0.31      0.11     0.09     0.51 1.00     4819
AOI2                    -0.91      0.11    -1.11    -0.70 1.00     3638
AOI3                    -0.30      0.11    -0.51    -0.08 1.00     5523
diagnosis1:emo1         -0.05      0.04    -0.14     0.04 1.00     9391
diagnosis2:emo1         -0.02      0.04    -0.11     0.07 1.00     9517
diagnosis3:emo1          0.10      0.04     0.02     0.18 1.00     9639
diagnosis1:emo2         -0.03      0.05    -0.12     0.06 1.00     8883
diagnosis2:emo2         -0.03      0.05    -0.12     0.06 1.00     8373
diagnosis3:emo2          0.00      0.04    -0.08     0.08 1.00    10071
diagnosis1:emo3          0.09      0.06    -0.02     0.20 1.00     7707
diagnosis2:emo3         -0.07      0.05    -0.17     0.04 1.00     6533
diagnosis3:emo3         -0.04      0.05    -0.14     0.05 1.00     8065
diagnosis1:AOI1          0.03      0.13    -0.24     0.28 1.00     7888
diagnosis2:AOI1         -0.03      0.13    -0.30     0.23 1.00     6633
diagnosis3:AOI1          0.15      0.13    -0.10     0.40 1.00     7037
diagnosis1:AOI2          0.04      0.13    -0.21     0.30 1.00     5114
diagnosis2:AOI2         -0.11      0.13    -0.37     0.14 1.00     5875
diagnosis3:AOI2          0.00      0.12    -0.23     0.25 1.00     5675
diagnosis1:AOI3         -0.07      0.14    -0.34     0.21 1.00     7152
diagnosis2:AOI3          0.04      0.13    -0.23     0.30 1.00     7119
diagnosis3:AOI3         -0.04      0.13    -0.29     0.23 1.00     7475
emo1:AOI1               -0.05      0.04    -0.13     0.03 1.00     9766
emo2:AOI1                0.23      0.04     0.15     0.31 1.00    10160
emo3:AOI1               -0.60      0.04    -0.69    -0.52 1.00     9570
emo1:AOI2               -0.09      0.05    -0.18     0.00 1.00     8816
emo2:AOI2                0.24      0.05     0.15     0.33 1.00     8886
emo3:AOI2               -0.52      0.05    -0.61    -0.42 1.00     7208
emo1:AOI3                0.28      0.04     0.19     0.36 1.00     8713
emo2:AOI3               -0.39      0.04    -0.47    -0.30 1.00     7537
emo3:AOI3                0.75      0.04     0.66     0.83 1.00     7983
diagnosis1:emo1:AOI1     0.02      0.07    -0.12     0.15 1.00     9490
diagnosis2:emo1:AOI1     0.04      0.07    -0.10     0.17 1.00     8450
diagnosis3:emo1:AOI1    -0.04      0.06    -0.16     0.08 1.00     9468
diagnosis1:emo2:AOI1    -0.05      0.07    -0.18     0.09 1.00     9501
diagnosis2:emo2:AOI1    -0.00      0.07    -0.13     0.13 1.00     8120
diagnosis3:emo2:AOI1     0.11      0.06    -0.01     0.23 1.00     9200
diagnosis1:emo3:AOI1     0.02      0.07    -0.12     0.16 1.00     8955
diagnosis2:emo3:AOI1     0.08      0.07    -0.05     0.22 1.00     7858
diagnosis3:emo3:AOI1    -0.07      0.06    -0.19     0.06 1.00     8265
diagnosis1:emo1:AOI2    -0.15      0.08    -0.30     0.00 1.00     8213
diagnosis2:emo1:AOI2     0.02      0.08    -0.13     0.18 1.00     7613
diagnosis3:emo1:AOI2     0.12      0.07    -0.01     0.25 1.00     8590
```

```
diagnosis1:emo2:AOI2     0.06      0.07    -0.08     0.20 1.00     8613
diagnosis2:emo2:AOI2    -0.12      0.07    -0.27     0.02 1.00     7574
diagnosis3:emo2:AOI2    -0.01      0.07    -0.15     0.12 1.00     8353
diagnosis1:emo3:AOI2     0.09      0.08    -0.07     0.24 1.00     8205
diagnosis2:emo3:AOI2    -0.16      0.08    -0.32     0.00 1.00     6300
diagnosis3:emo3:AOI2    -0.05      0.07    -0.19     0.09 1.00     8439
diagnosis1:emo1:AOI3     0.08      0.07    -0.06     0.22 1.00     9296
diagnosis2:emo1:AOI3    -0.04      0.07    -0.18     0.10 1.00     8339
diagnosis3:emo1:AOI3    -0.08      0.07    -0.21     0.05 1.00     7858
diagnosis1:emo2:AOI3    -0.15      0.07    -0.30    -0.01 1.00     9410
diagnosis2:emo2:AOI3     0.08      0.07    -0.06     0.22 1.00     7994
diagnosis3:emo2:AOI3     0.00      0.07    -0.12     0.13 1.00     8104
diagnosis1:emo3:AOI3     0.07      0.07    -0.07     0.22 1.00     8513
diagnosis2:emo3:AOI3     0.01      0.07    -0.13     0.15 1.00     7634
diagnosis3:emo3:AOI3     0.03      0.07    -0.10     0.16 1.00     7943
                     Tail_ESS
Intercept                3437
diagnosis1               3873
diagnosis2               4656
diagnosis3               3900
emo1                     6168
emo2                     6154
emo3                     6663
AOI1                     5409
AOI2                     5266
AOI3                     4972
diagnosis1:emo1          6221
diagnosis2:emo1          5830
diagnosis3:emo1          6753
diagnosis1:emo2          6572
diagnosis2:emo2          6221
diagnosis3:emo2          6442
diagnosis1:emo3          6535
diagnosis2:emo3          5942
diagnosis3:emo3          6534
diagnosis1:AOI1          5916
diagnosis2:AOI1          6361
diagnosis3:AOI1          5652
diagnosis1:AOI2          5631
diagnosis2:AOI2          6175
diagnosis3:AOI2          5588
diagnosis1:AOI3          5532
diagnosis2:AOI3          5772
diagnosis3:AOI3          6081
emo1:AOI1                6810
emo2:AOI1                6103
emo3:AOI1                6953
emo1:AOI2                6435
emo2:AOI2                6645
emo3:AOI2                6550
emo1:AOI3                6376
emo2:AOI3                5897
emo3:AOI3                6315
diagnosis1:emo1:AOI1     6455
diagnosis2:emo1:AOI1     6634
diagnosis3:emo1:AOI1     6643
diagnosis1:emo2:AOI1     6788
diagnosis2:emo2:AOI1     6333
diagnosis3:emo2:AOI1     6224
diagnosis1:emo3:AOI1     6934
```

```
diagnosis2:emo3:AOI1     6967
diagnosis3:emo3:AOI1     6444
diagnosis1:emo1:AOI2     6201
diagnosis2:emo1:AOI2     6644
diagnosis3:emo1:AOI2     6698
diagnosis1:emo2:AOI2     6053
diagnosis2:emo2:AOI2     6478
diagnosis3:emo2:AOI2     5687
diagnosis1:emo3:AOI2     6644
diagnosis2:emo3:AOI2     5897
diagnosis3:emo3:AOI2     6529
diagnosis1:emo1:AOI3     6068
diagnosis2:emo1:AOI3     6624
diagnosis3:emo1:AOI3     6930
diagnosis1:emo2:AOI3     6782
diagnosis2:emo2:AOI3     6356
diagnosis3:emo2:AOI3     7131
diagnosis1:emo3:AOI3     5811
diagnosis2:emo3:AOI3     6439
diagnosis3:emo3:AOI3     6217

Further Distributional Parameters:
      Estimate Est.Error l-95% CI u-95% CI Rhat Bulk_ESS Tail_ESS
sigma     0.47      0.01     0.45     0.50 1.00     4074     5603
hu        0.11      0.01     0.09     0.12 1.00    22063     5207

Draws were sampled using sample(hmc). For each parameter, Bulk_ESS
and Tail_ESS are effective sample size measures, and Rhat is the potential
scale reduction factor on split chains (at convergence, Rhat = 1).
```

6.2 Model summary for saccades

```
[1] "Contrast settings for Emotion"

   [,1] [,2] [,3]
AF    1    0    0
AN    0    1    0
HA    0    0    1
SA   -1   -1   -1

[1] "Contrast settings for Diagnosis"

     [,1] [,2] [,3]
ADHD    1    0    0
ASD     0    1    0
BOTH    0    0    1
COMP   -1   -1   -1

 Family: gaussian
  Links: mu = identity; sigma = identity
Formula: n.sac ~ diagnosis * emo + (1 | subID)
   Data: df.sac (Number of observations: 308)
  Draws: 4 chains, each with iter = 3000; warmup = 1000; thin = 1;
         total post-warmup draws = 8000

Multilevel Hyperparameters:
~subID (Number of levels: 77)
              Estimate Est.Error l-95% CI u-95% CI Rhat Bulk_ESS Tail_ESS
```

```
sd(Intercept)     1.37      0.09     1.20     1.55 1.00     2687     3601

Regression Coefficients:
                Estimate Est.Error l-95% CI u-95% CI Rhat Bulk_ESS Tail_ESS
Intercept           4.08      0.16     3.77     4.42 1.00     1725     2845
diagnosis1          0.12      0.29    -0.45     0.67 1.00     1777     2793
diagnosis2         -0.08      0.28    -0.65     0.46 1.00     1711     2883
diagnosis3          0.14      0.26    -0.39     0.64 1.00     1670     2663
emo1                0.31      0.08     0.16     0.47 1.00     8533     6236
emo2               -0.55      0.08    -0.70    -0.39 1.00     8532     6104
emo3                0.44      0.08     0.28     0.60 1.00     8497     6138
diagnosis1:emo1     0.03      0.14    -0.25     0.31 1.00     7333     5322
diagnosis2:emo1    -0.01      0.14    -0.26     0.26 1.00     7875     6051
diagnosis3:emo1     0.19      0.13    -0.06     0.45 1.00     6833     5466
diagnosis1:emo2     0.16      0.14    -0.11     0.43 1.00     7770     6520
diagnosis2:emo2    -0.04      0.14    -0.31     0.23 1.00     7758     5874
diagnosis3:emo2    -0.24      0.13    -0.49     0.02 1.00     7445     5752
diagnosis1:emo3     0.06      0.14    -0.23     0.34 1.00     7564     6218
diagnosis2:emo3    -0.10      0.14    -0.37     0.18 1.00     7602     6115
diagnosis3:emo3     0.25      0.13    -0.01     0.51 1.00     6962     5841

Further Distributional Parameters:
      Estimate Est.Error l-95% CI u-95% CI Rhat Bulk_ESS Tail_ESS
sigma     0.80      0.04     0.73     0.88 1.00     6015     5691

Draws were sampled using sample(hmc). For each parameter, Bulk_ESS
and Tail_ESS are effective sample size measures, and Rhat is the potential
scale reduction factor on split chains (at convergence, Rhat = 1).
```

7 First and last fixation

7.1 Model summary for first fixations

```
[1] "Coding of AOIs"

    AOI AOI.code
1  fore        2
2  nose        1
3  eyes        3
4 mouth        4

[1] "Contrast settings for Diagnosis"

     [,1] [,2] [,3]
ADHD    1    0    0
ASD     0    1    0
BOTH    0    0    1
COMP   -1   -1   -1

 Family: categorical
  Links: mu2 = logit; mu3 = logit; mu4 = logit
Formula: AOI.code ~ diagnosis + (1 | subID) + (diagnosis | video)
   Data: df.first (Number of observations: 4432)
  Draws: 4 chains, each with iter = 3000; warmup = 1000; thin = 1;
         total post-warmup draws = 8000

Multilevel Hyperparameters:
```

```
~subID (Number of levels: 77)
                  Estimate Est.Error l-95% CI u-95% CI Rhat Bulk_ESS Tail_ESS
sd(mu2_Intercept)     2.57      0.27     2.11     3.16 1.00     3535     5268
sd(mu3_Intercept)     2.19      0.19     1.84     2.61 1.00     2768     5084
sd(mu4_Intercept)     1.34      0.18     1.03     1.72 1.00     3101     5142

~video (Number of levels: 64)
                                   Estimate Est.Error l-95% CI u-95% CI Rhat
sd(mu2_Intercept)                      0.23      0.11     0.02     0.44 1.00
sd(mu2_diagnosis1)                     0.19      0.13     0.01     0.48 1.00
sd(mu2_diagnosis2)                     0.25      0.16     0.01     0.59 1.00
sd(mu2_diagnosis3)                     0.16      0.11     0.01     0.40 1.00
sd(mu3_Intercept)                      0.29      0.06     0.16     0.42 1.00
sd(mu3_diagnosis1)                     0.11      0.08     0.00     0.30 1.00
sd(mu3_diagnosis2)                     0.10      0.07     0.00     0.27 1.00
sd(mu3_diagnosis3)                     0.08      0.06     0.00     0.24 1.00
sd(mu4_Intercept)                      0.51      0.10     0.33     0.72 1.00
sd(mu4_diagnosis1)                     0.18      0.13     0.01     0.48 1.00
sd(mu4_diagnosis2)                     0.18      0.13     0.01     0.47 1.00
sd(mu4_diagnosis3)                     0.17      0.12     0.01     0.45 1.00
cor(mu2_Intercept,mu2_diagnosis1)     -0.05      0.37    -0.72     0.66 1.00
cor(mu2_Intercept,mu2_diagnosis2)     -0.04      0.37    -0.72     0.66 1.00
cor(mu2_diagnosis1,mu2_diagnosis2)    -0.00      0.38    -0.70     0.70 1.00
cor(mu2_Intercept,mu2_diagnosis3)     -0.04      0.37    -0.72     0.68 1.00
cor(mu2_diagnosis1,mu2_diagnosis3)    -0.06      0.38    -0.73     0.68 1.00
cor(mu2_diagnosis2,mu2_diagnosis3)    -0.04      0.37    -0.72     0.67 1.00
cor(mu3_Intercept,mu3_diagnosis1)      0.11      0.36    -0.62     0.74 1.00
cor(mu3_Intercept,mu3_diagnosis2)     -0.04      0.36    -0.71     0.65 1.00
cor(mu3_diagnosis1,mu3_diagnosis2)    -0.06      0.38    -0.74     0.67 1.00
cor(mu3_Intercept,mu3_diagnosis3)      0.03      0.36    -0.67     0.70 1.00
cor(mu3_diagnosis1,mu3_diagnosis3)    -0.09      0.40    -0.78     0.69 1.00
cor(mu3_diagnosis2,mu3_diagnosis3)    -0.04      0.38    -0.74     0.69 1.00
cor(mu4_Intercept,mu4_diagnosis1)      0.06      0.36    -0.65     0.71 1.00
cor(mu4_Intercept,mu4_diagnosis2)      0.05      0.35    -0.64     0.70 1.00
cor(mu4_diagnosis1,mu4_diagnosis2)    -0.09      0.38    -0.75     0.64 1.00
cor(mu4_Intercept,mu4_diagnosis3)      0.04      0.35    -0.64     0.68 1.00
cor(mu4_diagnosis1,mu4_diagnosis3)    -0.07      0.39    -0.76     0.67 1.00
cor(mu4_diagnosis2,mu4_diagnosis3)    -0.03      0.37    -0.71     0.69 1.00
                                   Bulk_ESS Tail_ESS
sd(mu2_Intercept)                      1833     3201
sd(mu2_diagnosis1)                     3803     4658
sd(mu2_diagnosis2)                     2858     4009
sd(mu2_diagnosis3)                     3405     4678
sd(mu3_Intercept)                      3161     4174
sd(mu3_diagnosis1)                     3605     4391
sd(mu3_diagnosis2)                     4737     4523
sd(mu3_diagnosis3)                     4626     4617
sd(mu4_Intercept)                      3834     5885
sd(mu4_diagnosis1)                     4193     4563
sd(mu4_diagnosis2)                     3311     5127
sd(mu4_diagnosis3)                     3009     4333
cor(mu2_Intercept,mu2_diagnosis1)     10352     6070
cor(mu2_Intercept,mu2_diagnosis2)      8927     6398
cor(mu2_diagnosis1,mu2_diagnosis2)     7159     6179
cor(mu2_Intercept,mu2_diagnosis3)     10626     6051
cor(mu2_diagnosis1,mu2_diagnosis3)     9015     6900
cor(mu2_diagnosis2,mu2_diagnosis3)     8098     6879
cor(mu3_Intercept,mu3_diagnosis1)     11806     5680
cor(mu3_Intercept,mu3_diagnosis2)     16128     5538
cor(mu3_diagnosis1,mu3_diagnosis2)    10101     6946
```

```
cor(mu3_Intercept,mu3_diagnosis3)     15470     5943
cor(mu3_diagnosis1,mu3_diagnosis3)     9737     6354
cor(mu3_diagnosis2,mu3_diagnosis3)     8763     7179
cor(mu4_Intercept,mu4_diagnosis1)     13739     6399
cor(mu4_Intercept,mu4_diagnosis2)     12788     6376
cor(mu4_diagnosis1,mu4_diagnosis2)     7942     6645
cor(mu4_Intercept,mu4_diagnosis3)     12346     6451
cor(mu4_diagnosis1,mu4_diagnosis3)     8303     6934
cor(mu4_diagnosis2,mu4_diagnosis3)     7968     6910

Regression Coefficients:
               Estimate Est.Error l-95% CI u-95% CI Rhat Bulk_ESS Tail_ESS
mu2_Intercept     -2.21      0.32    -2.85    -1.58 1.00     2099     3221
mu3_Intercept     -0.47      0.27    -1.00     0.05 1.00     1360     2917
mu4_Intercept     -2.19      0.21    -2.61    -1.80 1.00     3391     5265
mu2_diagnosis1     0.28      0.49    -0.69     1.24 1.00     1910     3709
mu2_diagnosis2    -0.71      0.50    -1.68     0.26 1.00     2977     4622
mu2_diagnosis3     0.48      0.45    -0.42     1.37 1.00     1913     3636
mu3_diagnosis1     0.27      0.42    -0.57     1.07 1.00     1852     2990
mu3_diagnosis2    -0.14      0.41    -0.92     0.65 1.00     1594     2816
mu3_diagnosis3     0.12      0.38    -0.63     0.85 1.00     1751     3058
mu4_diagnosis1     0.01      0.31    -0.61     0.63 1.00     3976     5276
mu4_diagnosis2     0.03      0.31    -0.57     0.64 1.00     3394     4507
mu4_diagnosis3    -0.24      0.29    -0.82     0.32 1.00     3641     4080

Draws were sampled using sample(hmc). For each parameter, Bulk_ESS
and Tail_ESS are effective sample size measures, and Rhat is the potential
scale reduction factor on split chains (at convergence, Rhat = 1).
```

7.2 Model summary for last fixations

```
[1] "Coding of AOIs"

    AOI AOI.code
1  fore        2
2  nose        1
3  eyes        3
4 mouth        4

[1] "Contrast settings for Emotion"

   [,1] [,2] [,3]
AF    1    0    0
AN    0    1    0
HA    0    0    1
SA   -1   -1   -1

[1] "Contrast settings for Diagnosis"

     [,1] [,2] [,3]
ADHD    1    0    0
ASD     0    1    0
BOTH    0    0    1
COMP   -1   -1   -1
```

```
 Family: categorical
  Links: mu2 = logit; mu3 = logit; mu4 = logit
Formula: AOI.code ~ diagnosis * emo + (emo | subID) + (diagnosis | video)
   Data: df.last (Number of observations: 3511)
  Draws: 4 chains, each with iter = 3000; warmup = 1000; thin = 1;
         total post-warmup draws = 8000

Multilevel Hyperparameters:
~subID (Number of levels: 75)
                            Estimate Est.Error l-95% CI u-95% CI Rhat Bulk_ESS
sd(mu2_Intercept)               1.94      0.26     1.49     2.51 1.00     4517
sd(mu2_emo1)                    0.36      0.25     0.02     0.92 1.00     3885
sd(mu2_emo2)                    0.39      0.24     0.02     0.91 1.00     2724
sd(mu2_emo3)                    0.58      0.28     0.06     1.13 1.00     2686
sd(mu3_Intercept)               1.76      0.17     1.45     2.13 1.00     4006
sd(mu3_emo1)                    0.12      0.09     0.00     0.34 1.00     5128
sd(mu3_emo2)                    0.22      0.13     0.01     0.48 1.00     3071
sd(mu3_emo3)                    0.40      0.14     0.10     0.65 1.00     2380
sd(mu4_Intercept)               1.79      0.19     1.45     2.20 1.00     3744
sd(mu4_emo1)                    0.16      0.12     0.01     0.44 1.00     4094
sd(mu4_emo2)                    0.19      0.13     0.01     0.50 1.00     3754
sd(mu4_emo3)                    0.53      0.15     0.22     0.82 1.00     2583
cor(mu2_Intercept,mu2_emo1)     0.19      0.35    -0.56     0.77 1.00    12205
cor(mu2_Intercept,mu2_emo2)     0.19      0.33    -0.50     0.76 1.00    11612
cor(mu2_emo1,mu2_emo2)         -0.09      0.37    -0.73     0.66 1.00     6125
cor(mu2_Intercept,mu2_emo3)    -0.22      0.30    -0.74     0.40 1.00    10167
cor(mu2_emo1,mu2_emo3)          0.01      0.36    -0.67     0.70 1.00     5220
cor(mu2_emo2,mu2_emo3)         -0.23      0.36    -0.81     0.56 1.00     4228
cor(mu3_Intercept,mu3_emo1)     0.05      0.37    -0.67     0.72 1.00    17315
cor(mu3_Intercept,mu3_emo2)    -0.16      0.32    -0.73     0.51 1.00    12362
cor(mu3_emo1,mu3_emo2)         -0.11      0.38    -0.78     0.65 1.00     5435
cor(mu3_Intercept,mu3_emo3)     0.07      0.26    -0.43     0.56 1.00    11336
cor(mu3_emo1,mu3_emo3)         -0.09      0.37    -0.75     0.64 1.00     2633
cor(mu3_emo2,mu3_emo3)         -0.01      0.35    -0.66     0.66 1.00     3442
cor(mu4_Intercept,mu4_emo1)    -0.04      0.36    -0.71     0.66 1.00    14984
cor(mu4_Intercept,mu4_emo2)     0.14      0.36    -0.60     0.74 1.00    14174
cor(mu4_emo1,mu4_emo2)         -0.02      0.37    -0.71     0.70 1.00     8314
cor(mu4_Intercept,mu4_emo3)    -0.41      0.21    -0.75     0.05 1.00    10503
cor(mu4_emo1,mu4_emo3)         -0.09      0.37    -0.75     0.64 1.00     2341
cor(mu4_emo2,mu4_emo3)         -0.22      0.37    -0.81     0.58 1.00     2327
                            Tail_ESS
sd(mu2_Intercept)               5753
sd(mu2_emo1)                    5103
sd(mu2_emo2)                    4085
sd(mu2_emo3)                    3411
sd(mu3_Intercept)               5379
sd(mu3_emo1)                    5309
sd(mu3_emo2)                    4100
sd(mu3_emo3)                    1730
sd(mu4_Intercept)               5114
sd(mu4_emo1)                    5080
sd(mu4_emo2)                    4912
sd(mu4_emo3)                    3118
cor(mu2_Intercept,mu2_emo1)     6000
cor(mu2_Intercept,mu2_emo2)     6409
cor(mu2_emo1,mu2_emo2)          6411
cor(mu2_Intercept,mu2_emo3)     6263
cor(mu2_emo1,mu2_emo3)          6704
cor(mu2_emo2,mu2_emo3)          5676
cor(mu3_Intercept,mu3_emo1)     5874
```

```
cor(mu3_Intercept,mu3_emo2)     6159
cor(mu3_emo1,mu3_emo2)          5934
cor(mu3_Intercept,mu3_emo3)     5649
cor(mu3_emo1,mu3_emo3)          4455
cor(mu3_emo2,mu3_emo3)          4666
cor(mu4_Intercept,mu4_emo1)     5317
cor(mu4_Intercept,mu4_emo2)     6379
cor(mu4_emo1,mu4_emo2)          6467
cor(mu4_Intercept,mu4_emo3)     5715
cor(mu4_emo1,mu4_emo3)          4142
cor(mu4_emo2,mu4_emo3)          4430

~video (Number of levels: 64)
                                   Estimate Est.Error l-95% CI u-95% CI Rhat
sd(mu2_Intercept)                      0.41      0.18     0.05     0.76 1.00
sd(mu2_diagnosis1)                     0.31      0.21     0.01     0.75 1.00
sd(mu2_diagnosis2)                     0.31      0.21     0.02     0.79 1.00
sd(mu2_diagnosis3)                     0.24      0.17     0.01     0.63 1.00
sd(mu3_Intercept)                      0.39      0.07     0.26     0.55 1.00
sd(mu3_diagnosis1)                     0.14      0.10     0.00     0.38 1.00
sd(mu3_diagnosis2)                     0.10      0.08     0.00     0.30 1.00
sd(mu3_diagnosis3)                     0.19      0.11     0.01     0.42 1.00
sd(mu4_Intercept)                      0.42      0.08     0.27     0.60 1.00
sd(mu4_diagnosis1)                     0.16      0.12     0.01     0.44 1.00
sd(mu4_diagnosis2)                     0.14      0.10     0.00     0.38 1.00
sd(mu4_diagnosis3)                     0.26      0.13     0.02     0.52 1.00
cor(mu2_Intercept,mu2_diagnosis1)      0.11      0.37    -0.62     0.76 1.00
cor(mu2_Intercept,mu2_diagnosis2)      0.10      0.37    -0.63     0.75 1.00
cor(mu2_diagnosis1,mu2_diagnosis2)    -0.05      0.38    -0.73     0.69 1.00
cor(mu2_Intercept,mu2_diagnosis3)      0.06      0.37    -0.65     0.73 1.00
cor(mu2_diagnosis1,mu2_diagnosis3)    -0.01      0.37    -0.70     0.69 1.00
cor(mu2_diagnosis2,mu2_diagnosis3)    -0.00      0.38    -0.71     0.71 1.00
cor(mu3_Intercept,mu3_diagnosis1)      0.06      0.36    -0.64     0.71 1.00
cor(mu3_Intercept,mu3_diagnosis2)      0.06      0.37    -0.64     0.72 1.00
cor(mu3_diagnosis1,mu3_diagnosis2)    -0.06      0.38    -0.74     0.67 1.00
cor(mu3_Intercept,mu3_diagnosis3)     -0.39      0.32    -0.86     0.36 1.00
cor(mu3_diagnosis1,mu3_diagnosis3)    -0.18      0.38    -0.81     0.60 1.00
cor(mu3_diagnosis2,mu3_diagnosis3)    -0.13      0.38    -0.79     0.62 1.00
cor(mu4_Intercept,mu4_diagnosis1)      0.04      0.35    -0.64     0.70 1.00
cor(mu4_Intercept,mu4_diagnosis2)      0.06      0.36    -0.66     0.72 1.00
cor(mu4_diagnosis1,mu4_diagnosis2)    -0.09      0.38    -0.77     0.65 1.00
cor(mu4_Intercept,mu4_diagnosis3)      0.23      0.30    -0.42     0.75 1.00
cor(mu4_diagnosis1,mu4_diagnosis3)    -0.12      0.38    -0.77     0.62 1.00
cor(mu4_diagnosis2,mu4_diagnosis3)    -0.02      0.38    -0.72     0.70 1.00
                                   Bulk_ESS Tail_ESS
sd(mu2_Intercept)                      1912     2676
sd(mu2_diagnosis1)                     3533     4174
sd(mu2_diagnosis2)                     3775     4646
sd(mu2_diagnosis3)                     3906     5296
sd(mu3_Intercept)                      3795     5049
sd(mu3_diagnosis1)                     3948     5035
sd(mu3_diagnosis2)                     5381     5043
sd(mu3_diagnosis3)                     2944     4121
sd(mu4_Intercept)                      3839     5195
sd(mu4_diagnosis1)                     4663     5462
sd(mu4_diagnosis2)                     4639     4455
sd(mu4_diagnosis3)                     2452     3492
cor(mu2_Intercept,mu2_diagnosis1)      8709     5730
cor(mu2_Intercept,mu2_diagnosis2)     10938     6516
cor(mu2_diagnosis1,mu2_diagnosis2)     9350     6717
```

```
cor(mu2_Intercept,mu2_diagnosis3)     11263     6712
cor(mu2_diagnosis1,mu2_diagnosis3)     9080     6704
cor(mu2_diagnosis2,mu2_diagnosis3)     7679     6564
cor(mu3_Intercept,mu3_diagnosis1)     11978     6500
cor(mu3_Intercept,mu3_diagnosis2)     12862     6217
cor(mu3_diagnosis1,mu3_diagnosis2)    11503     6364
cor(mu3_Intercept,mu3_diagnosis3)      7237     6408
cor(mu3_diagnosis1,mu3_diagnosis3)     6439     6352
cor(mu3_diagnosis2,mu3_diagnosis3)     7068     6482
cor(mu4_Intercept,mu4_diagnosis1)     14241     6260
cor(mu4_Intercept,mu4_diagnosis2)     15011     6629
cor(mu4_diagnosis1,mu4_diagnosis2)     9695     6199
cor(mu4_Intercept,mu4_diagnosis3)      9075     6467
cor(mu4_diagnosis1,mu4_diagnosis3)     5204     5735
cor(mu4_diagnosis2,mu4_diagnosis3)     6283     6479

Regression Coefficients:
                     Estimate Est.Error l-95% CI u-95% CI Rhat Bulk_ESS Tail_ESS
mu2_Intercept           -3.26      0.32    -3.93    -2.68 1.00     5081     4976
mu3_Intercept           -0.45      0.23    -0.89    -0.01 1.00     2666     4358
mu4_Intercept           -1.29      0.23    -1.76    -0.84 1.00     3441     5240
mu2_diagnosis1          -0.02      0.44    -0.90     0.83 1.00     5775     6144
mu2_diagnosis2          -0.54      0.47    -1.46     0.38 1.00     6532     6216
mu2_diagnosis3           0.08      0.40    -0.71     0.86 1.00     4969     5928
mu2_emo1                 0.05      0.27    -0.53     0.55 1.00     9128     6477
mu2_emo2                 0.54      0.24     0.05     0.99 1.00     8303     6305
mu2_emo3                -0.46      0.27    -0.98     0.07 1.00     9927     6535
mu2_diagnosis1:emo1     -0.16      0.35    -0.86     0.53 1.00    12555     5779
mu2_diagnosis2:emo1     -0.02      0.41    -0.84     0.74 1.00    12056     6484
mu2_diagnosis3:emo1      0.03      0.31    -0.58     0.60 1.00    11496     6746
mu2_diagnosis1:emo2     -0.21      0.30    -0.79     0.37 1.00    11406     6749
mu2_diagnosis2:emo2      0.10      0.34    -0.57     0.77 1.00    12788     6973
mu2_diagnosis3:emo2      0.16      0.27    -0.37     0.70 1.00    11701     6364
mu2_diagnosis1:emo3      0.24      0.34    -0.43     0.89 1.00    11349     6520
mu2_diagnosis2:emo3      0.18      0.38    -0.57     0.91 1.00    12702     6713
mu2_diagnosis3:emo3     -0.53      0.33    -1.20     0.08 1.00    10913     6319
mu3_diagnosis1          -0.02      0.36    -0.73     0.69 1.00     2672     3979
mu3_diagnosis2          -0.14      0.37    -0.86     0.58 1.00     3331     4922
mu3_diagnosis3           0.50      0.33    -0.14     1.15 1.00     2355     4119
mu3_emo1                 0.35      0.13     0.10     0.61 1.00     9651     6575
mu3_emo2                 0.16      0.13    -0.08     0.41 1.00     9700     6778
mu3_emo3                -0.88      0.14    -1.16    -0.61 1.00     9548     6425
mu3_diagnosis1:emo1      0.09      0.17    -0.24     0.43 1.00    11776     6670
mu3_diagnosis2:emo1     -0.01      0.19    -0.39     0.37 1.00    10707     6327
mu3_diagnosis3:emo1      0.10      0.16    -0.20     0.41 1.00     9694     6663
mu3_diagnosis1:emo2     -0.18      0.16    -0.49     0.14 1.00    11428     6593
mu3_diagnosis2:emo2     -0.01      0.18    -0.36     0.33 1.00    11717     6857
mu3_diagnosis3:emo2     -0.00      0.15    -0.29     0.29 1.00    11293     6061
mu3_diagnosis1:emo3      0.15      0.18    -0.21     0.51 1.00    11492     6512
mu3_diagnosis2:emo3     -0.29      0.20    -0.69     0.10 1.00    11448     5927
mu3_diagnosis3:emo3     -0.00      0.17    -0.33     0.33 1.00     9337     6671
mu4_diagnosis1          -0.22      0.38    -0.96     0.51 1.00     3075     4389
mu4_diagnosis2          -0.03      0.38    -0.77     0.70 1.00     3685     4899
mu4_diagnosis3          -0.01      0.35    -0.69     0.67 1.00     2977     4455
mu4_emo1                 0.48      0.15     0.19     0.78 1.00    10453     6573
mu4_emo2                -0.57      0.16    -0.88    -0.27 1.00    10196     7210
mu4_emo3                 0.62      0.16     0.32     0.92 1.00     7499     6410
mu4_diagnosis1:emo1      0.04      0.20    -0.35     0.43 1.00    12475     7098
mu4_diagnosis2:emo1      0.32      0.20    -0.08     0.72 1.00    11502     6557
mu4_diagnosis3:emo1     -0.32      0.19    -0.70     0.06 1.00    10916     6463
```

```
mu4_diagnosis1:emo2    -0.44      0.22    -0.88    -0.01 1.00    12248     6812
mu4_diagnosis2:emo2    -0.30      0.21    -0.73     0.12 1.00    11109     6333
mu4_diagnosis3:emo2     0.48      0.19     0.11     0.86 1.00    11132     6496
mu4_diagnosis1:emo3     0.24      0.21    -0.16     0.65 1.00     8997     6156
mu4_diagnosis2:emo3    -0.30      0.21    -0.72     0.14 1.00     9675     6243
mu4_diagnosis3:emo3     0.11      0.20    -0.28     0.49 1.00     9619     6496

Draws were sampled using sample(hmc). For each parameter, Bulk_ESS
and Tail_ESS are effective sample size measures, and Rhat is the potential
scale reduction factor on split chains (at convergence, Rhat = 1).
```
